# Virtual single‐cell perturbation and genetic causal inference reveal *CSF1R*‐dependent immunometabolic communication in iron metabolism‐associated osteoarthritis

**DOI:** 10.1002/ccs3.70107

**Published:** 2026-08-27

**Authors:** Yue Zhou, Guohang Shen, Lijing Si, Kaiyong Wang, Yang Chen, Ruoyan Wang, Yupei Dai

**Affiliations:** ^1^ Affiliated Hospital of North Sichuan Medical College Nanchong Sichuan China; ^2^ North Sichuan Medical College Nanchong Sichuan China; ^3^ Department of Clinical Medicine Ningxia Medical University Yinchuan China; ^4^ School of Basic Medicine and Forensic Medicine North Sichuan Medical College Nanchong Sichuan China

**Keywords:** iron metabolism disorder, macrophage, Mendelian randomization, osteoarthritis, single‐cell RNA sequencing

## Abstract

Iron dysregulation has emerged as a contributor to osteoarthritis (OA), yet the cell‐communication mechanisms connecting iron‐related genetic signals to joint degeneration remain insufficiently defined. Here, we established an integrative computational framework combining transcriptomic screening, machine learning, Mendelian randomization, immune and metabolite mediation analysis, single‐cell transcriptomics, virtual gene perturbation, molecular docking, molecular dynamics simulation, and experimental validation to identify signaling regulators involved in iron metabolism‐associated OA. By intersecting iron metabolism‐related genes, OA differentially expressed genes, and eQTL‐supported genes, we identified 27 shared candidates. Machine learning‐based prioritization and genetic causal inference further highlighted *CSF1R* as a central regulatory gene. Single‐cell analysis localized CSF1R expression predominantly to macrophages, indicating a macrophage‐centered role in the osteoarthritic microenvironment. Mediation analysis integrating 731 immune‐cell traits and 1400 circulating metabolites identified CD14^+^CD16^+^ monocytes as a significant cellular mediator linking *CSF1R* activity to OA susceptibility, suggesting that *CSF1R* may promote disease progression mainly through monocyte–macrophage remodeling rather than isolated metabolic alteration. Genetic colocalization further supported a shared regulatory signal between *CSF1R* expression and OA risk. Virtual single‐cell perturbation revealed distinct downstream consequences of *CSF1R* modulation. Simulated *CSF1R* depletion enhanced antigen processing, major histocompatibility complex class II presentation, and phagosome‐related programs, whereas simulated *CSF1R* overexpression preferentially activated extracellular matrix organization, integrin signaling, and cartilage development‐associated pathways. Structural analyses identified stable interactions between *CSF1R* and candidate inhibitory compounds, and inflammatory stimulation of macrophages confirmed increased *CSF1R* protein expression. Collectively, this study identifies *CSF1R* as a macrophage‐associated immunometabolic signaling hub linking iron dysregulation to OA. These findings provide a mechanistic basis for targeting *CSF1R*‐mediated monocyte–macrophage communication and offer a computational strategy for prioritizing therapeutic targets in degenerative joint disease.

## Graphical abstract

Integrative genetic, single‐cell, and perturbation analyses identify *CSF1R* as a macrophage‐centered immunometabolic signaling hub linking iron dysregulation to osteoarthritis. *CSF1R*‐driven monocyte–macrophage remodeling may coordinate immune activation, extracellular matrix remodeling, and cartilage degeneration, providing a potential therapeutic target.

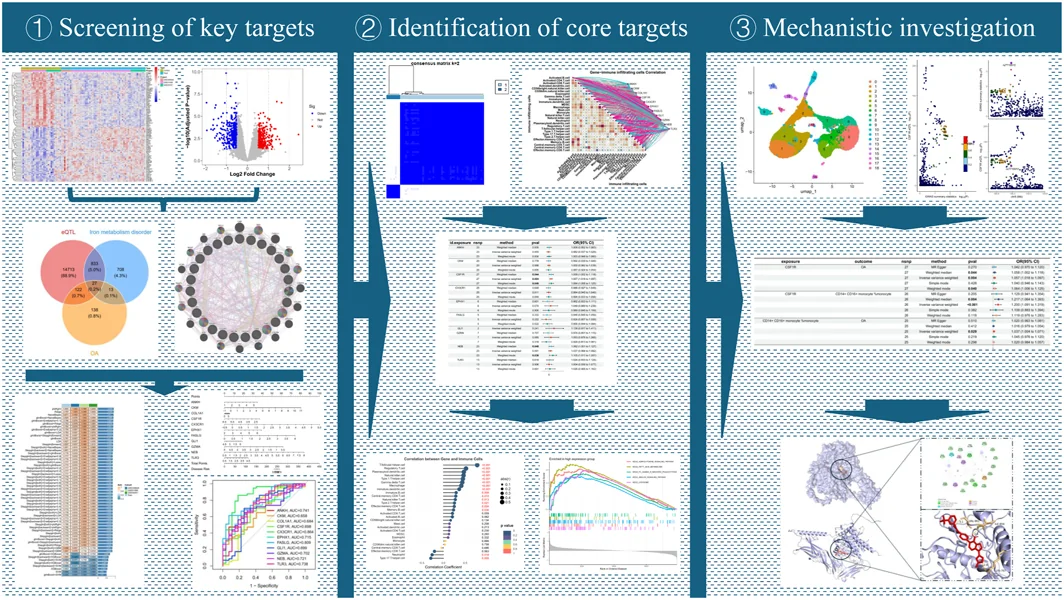

## 1 INTRODUCTION

Osteoarthritis (OA) is one of the leading causes of disability among middle‐aged and elderly individuals, affecting hundreds of millions of people worldwide. The principal pathological features of OA include synovial inflammation, subchondral bone sclerosis, and degradation of articular cartilage.^1^, ^2^ Current studies have demonstrated that dysregulation of immune cell function and intracellular iron metabolism play pivotal roles in the pathogenesis of OA. Specifically, synovial tissues from OA patients exhibit a significant increase in various immune cell populations, including plasma cells, M1 and M2 macrophages, activated dendritic cells, and resting mast cells.^3^, ^4^ Moreover, disturbed iron metabolism within knee joint cells leads to intracellular iron accumulation, which in turn promotes the generation of reactive oxygen species, triggers ferroptosis, and ultimately accelerates the progression of OA.^5^, ^6^, ^7^

Disruption of iron homeostasis, characterized by dysregulated iron absorption, transport, storage, and utilization, can lead to systemic or localized iron overload. Such imbalance not only perturbs cellular redox equilibrium and interferes with energy metabolism, but also profoundly influences immune cell differentiation, proliferation, and activation, thereby contributing to immune dysregulation.^8^, ^9^ Accumulating clinical and experimental evidence indicates a close association between aberrant iron metabolism and the initiation and progression of OA. In particular, excessive iron deposition within joint tissues has been shown to exacerbate cartilage degeneration and synovial inflammation.^10^ Despite these advances, the precise molecular mechanisms through which iron dysregulation drives OA pathology, as well as the identification of key regulatory targets, remain incompletely understood. Existing studies have largely focused on isolated pathological processes or individual signaling pathways, lacking a comprehensive, multi‐dimensional framework integrating genetic causality, cellular heterogeneity, and immunometabolic interactions. Moreover, reliable biomarkers for early diagnosis and targeted intervention in iron metabolism‐associated OA have yet to be established, posing a significant barrier to the development of precision therapeutic strategies.

The present study adopts an integrative, multi‐dimensional strategy to systematically investigate the intrinsic link between iron metabolism dysregulation and OA. By combining bioinformatics analyses, machine learning‐based modeling, Mendelian randomization (MR), single‐cell RNA sequencing, mediation analysis, genetic colocalization, molecular docking, and molecular dynamics (MD) simulations, we sought to identify key regulatory targets and delineate the molecular mechanisms underlying iron metabolism‐driven OA progression. Specifically, this work aims to define shared core targets connecting iron metabolism imbalance and OA, characterize their functional roles within immune cell regulation and metabolic pathway interactions, and evaluate their translational potential as therapeutic targets. Through this comprehensive framework, the study is expected to provide mechanistic insights into iron metabolism‐associated OA and establish a theoretical and experimental foundation for early detection, mechanistic interpretation, and targeted therapeutic development.

## 2 METHODS

### 2.1 Investigating the association between iron metabolism disorders and OA

To investigate the causal relationship between iron metabolism disorders and knee OA, MR analysis was performed using summary‐level genetic data. Genetic variants associated with iron metabolism‐related traits were obtained from the FinnGen consortium database. Single nucleotide polymorphisms (SNPs) were considered candidate instrumental variables (IVs) when they showed genome‐wide significant associations with the exposure (*p* < 5 × 10^−8^). To ensure independence among genetic instruments, linkage disequilibrium (LD) clumping was performed using a 1000 kb window and an *r* ^2^ threshold of 0.1. The remaining independent SNPs were subsequently used as IVs for MR analysis. Outcome data for OA were obtained from the GWAS database (GWAS ID: GCST007090).

### 2.2 Identification of shared target genes between iron metabolism disorder and OA

Candidate genes associated with iron metabolism disorders were retrieved from the GeneCards database. Three OA‐related transcriptomic datasets were downloaded from the GEO database (detailed in Supporting Information S1: Table S1). To minimize technical variability across datasets, batch effects were corrected using the sva R package, and the effectiveness of normalization was assessed via principal component analysis (PCA). Differential expression analysis was subsequently performed using the limma R package, with thresholds set at |log2 fold change (logFC)| > 0.5 and *p* < 0.05 to identify differentially expressed genes (DEGs) in OA.^11^ The intersection of (i) iron metabolism‐related genes, (ii) OA‐associated DEGs, and (iii) genes with expression quantitative trait loci (eQTL) data from the eQTLGen database was defined as the set of shared candidate target genes linking iron metabolism dysregulation to OA. Detailed database sources are provided in Supporting Information S1: Table S2.

### 2.3 Functional enrichment analysis and PPI network construction of shared genes

To explore the biological functions of the shared target genes, functional annotation and pathway enrichment analyses were conducted using the clusterProfiler, org.Hs.eg.db, and enrichplot R packages. Gene ontology (GO) and Kyoto encyclopedia of genes and genomes (KEGG) analyses were performed, with *p* < 0.05 considered statistically significant.

A protein–protein interaction (PPI) network was constructed using the GeneMANIA database to evaluate potential physical interactions and functional associations among the shared genes. This analysis facilitated the identification of key regulatory modules and provided insights into the molecular interaction landscape underlying iron metabolism‐associated OA.^12^, ^13^

### 2.4 Construction of predictive models

A total of 113 predictive models were constructed by combining 12 machine learning algorithms, including lasso, ridge, stepwise logistic regression (Stepglm), XGBoost, random forest, elastic net, plsRglm, glmBoost, and support vector machine, among others. The integrated dataset was randomly divided into a training set (70%) and a test set (30%), with specific parameters detailed in Supporting Information S1: Table S3. The model with the highest average area under the curve (AUC) values in both the training and test sets was selected as the optimal model. Model performance was further evaluated using nomograms, receiver operating characteristic (ROC) curves, and calibration curves.

### 2.5 Consensus clustering analysis

Based on the expression profiles of key genes identified from the diagnostic model, unsupervised hierarchical clustering was performed on OA samples using the “ConsensusClusterPlus” R package. Immune‐related mechanisms were analyzed using the “GSVA” and “GSEAbase” R packages to assess immune infiltration characteristics across different subgroups. In addition, the chromosomal locations of each key gene were visualized using the “circlize” package.

### 2.6 MR analysis

To further investigate the causal relationship between key genes and OA, expression quantitative trait loci (eQTL) data for the candidate genes were extracted from the eQTLGen database and used as exposure variables. Summary‐level outcome data for OA were retrieved from the GWAS database (GWAS ID: GCST007090), comprising 29,999,696 SNPs derived from 403,124 individuals, including 24,955 cases and 378,169 controls. MR analysis was subsequently performed using the “TwoSampleMR” R package to identify key targets that may exhibit a causal association with OA.^14^

### 2.7 Mediation analysis

A two‐step MR framework was employed to investigate whether the core target gene influences OA progression through immune cell traits and circulating metabolites. Summary‐level genetic data for 731 immune cell phenotypes (GWAS ID: GCST90001391–GCST90002121) and 1400 metabolites (GWAS ID: GCST90199621–GCST90201020) were obtained from publicly available GWAS datasets. IVs were selected using a significance threshold of *p* < 1 × 10^−5^. LD was controlled by applying clumping procedures with a window size of 10,000 kb and an *r* ^2^ threshold <0.001 to ensure independence among selected variants.

The mediation effect was quantified using the product of coefficients method, calculated as (*β*_1_ × *β*_2_)/*β*_total, where *β*_1_ represents the effect of the core gene on the mediator, *β*_2_ represents the effect of the mediator on OA, and *β*_total denotes the total effect of the core gene on OA. Immune cell subsets and metabolites exhibiting significant mediation effects were identified as potential intermediates linking the core gene to OA progression.^15^, ^16^

### 2.8 Single‐gene GSEA and single‐cell analysis

Gene set enrichment analysis (GSEA) was performed using the “org.Hs.eg.db” and “clusterProfiler” R packages, with gene sets obtained from the official database c2.cp.kegg.v7.3.symbols.ggt. Pearson correlation analysis was subsequently used to examine the associations between key genes and immune cell subtypes. Single‐cell RNA sequencing data for OA were retrieved from the GEO database (dataset ID: GSE248453). The dataset was processed and converted into a Seurat object using the “Seurat” R package. After quality control and data filtering, the top 2000 highly variable genes were selected for PCA. Uniform manifold approximation and projection (UMAP) was then employed for unsupervised clustering and unbiased visualization of cell subpopulations. Cell‐type annotation within each cluster was performed using the “SingleR” R package.^17^ Genes with log2FC > 1 and adjusted *p*‐value (*p*_val_adj) < 0.05 were defined as cluster‐specific marker genes.

### 2.9 Immune infiltration analysis

To assess the composition and infiltration characteristics of immune cells within the tissue microenvironment, the single‐sample gene set enrichment analysis (ssGSEA) algorithm was applied using the “limma,” “GSEABase,” and “GSVA” R packages. This method enables the estimation of the relative abundance of 28 distinct immune cell types.^18^ Furthermore, the “ggplot2” R package was utilized to analyze the correlation between the expression levels of the core gene and the degree of immune cell infiltration.

### 2.10 Co‐localization analysis

OA‐related genetic data were obtained from the GWAS database. Bayesian co‐localization analysis was performed using the “coloc” R package to identify shared causal variants between expression quantitative trait loci associated with the core gene and OA susceptibility. This analysis integrated summary‐level statistics from both the OA GWAS dataset and the eQTL dataset. A posterior probability (PPH4) indicating a high likelihood of shared causal variants between gene expression and OA was considered evidence of co‐localization.

### 2.11 Molecular docking

In this study, candidate drugs targeting the core gene were initially screened using the DSigDB database, and potentially toxic compounds were excluded based on literature review. To further analyze the interactions between the core target protein and candidate compounds, molecular docking was performed. The two‐dimensional (2D) structures of small‐molecule ligands were retrieved from the PubChem database and subsequently converted into three‐dimensional (3D) structures using Chem Office 20.0 software. The 3D structure of the core target protein was obtained from the RCSB Protein Data Bank, and the protein structure was preprocessed by removing water molecules and ligands, as well as adding hydrogen atoms. AutoDockTools 1.5.7 was used to further process the target protein and define the active binding pocket. Docking scores between small molecules and the core protein were then calculated using the Autodock Vina software. Finally, docking results were visualized using PyMOL software.

### 2.12 MD simulation

To further evaluate the binding stability between Kinome_439 and the core target protein, MD simulations were performed using GROMACS 2022. The Amber14SB force field was applied to the protein, while GAFF2 parameters were assigned to the ligand, with topology files generated using pdb2gmx and AutoFF.

The protein–ligand complex was solvated in a cubic TIP3P water box with a 1 nm buffer distance, and counterions were added to neutralize the system. Long‐range electrostatic interactions were calculated using the Particle Mesh Ewald method with a cutoff of 1 nm, and covalent bonds were constrained using the SHAKE algorithm with a 1 fs Verlet integration step. Prior to production simulation, energy minimization was conducted using a combination of steepest descent (3000 steps) and conjugate gradient (2000 steps) algorithms. Subsequently, a 100 ns MD simulation was carried out under NPT conditions (310 K, 1 atm). Structural stability and dynamic behavior of the complex were assessed by calculating root mean square deviation (RMSD), root mean square fluctuation (RMSF), hydrogen bond interactions, radius of gyration (Rg), and solvent‐accessible surface area (SASA). Binding free energy was further estimated using the MM‐PBSA method.

### 2.13 In vitro validation of the core target

The expression of the core target protein under inflammatory conditions was evaluated using Western blot (WB) analysis. RAW264.7 macrophages were maintained in Dulbecco's modified Eagle's medium supplemented with 10% fetal bovine serum and antibiotics under standard cell culture conditions (37°C, 5% CO_2_). To induce macrophage inflammatory activation, cells were stimulated with lipopolysaccharide for the indicated duration. Cells without stimulation were used as controls.

Total protein was extracted using RIPA lysis buffer supplemented with protease inhibitors, followed by centrifugation to obtain protein lysates. Protein concentrations were determined using a BCA assay. Equal amounts of protein were separated by SDS‐PAGE and transferred onto PVDF membranes. After blocking, membranes were incubated with primary antibodies against *CSF1R* at 4°C overnight, followed by incubation with HRP‐conjugated secondary antibodies at room temperature. Protein bands were visualized using enhanced chemiluminescence, and band intensities were quantified using an imaging system to compare *CSF1R* expression levels between experimental and control groups.

### 2.14 Single‐cell gene perturbation simulation analysis

To investigate the regulatory effects of the core gene *CSF1R* at single‐cell resolution, gene perturbation simulations were performed using the OA single‐cell transcriptomic dataset. Single‐cell gene regulatory networks were independently reconstructed using the scTenifoldKnk and scTenifoldOE algorithms to simulate loss‐of‐function (gene knockout) and gain‐of‐function (gene overexpression) perturbations of *CSF1R*, respectively. Regulatory network alterations induced by each perturbation were quantified through differential network analysis, and downstream genes exhibiting the most pronounced perturbation‐associated transcriptional responses were identified. Functional annotation of the responsive gene sets was subsequently conducted using GO enrichment and KEGG pathway analyses to characterize the biological processes and signaling pathways potentially governed by *CSF1R* in OA.

### 2.15 Statistical analysis

Statistical analyses were performed using R software (version 4.4.1) with the corresponding packages described in each analytical section. Continuous variables were presented as mean ± standard deviation or median with interquartile range according to data distribution. Comparisons between two groups were conducted using Student's *t*‐test or Wilcoxon rank‐sum test, as appropriate. Correlation analyses were performed using Pearson's or Spearman's correlation coefficients according to data characteristics. Differential expression analysis was conducted using the limma package, with |log2FC| > 0.5 and *p* < 0.05 considered significant. Pathway enrichment was considered statistically significant at *p* < 0.05. Machine learning model performance was assessed using AUC, calibration curves, and decision curve analysis (DCA). MR analyses were performed according to the criteria described above, with *p* < 0.05 considered statistically significant. Unless otherwise specified, all statistical tests were two‐sided. Statistical significance in figures was defined as follows: ns, *p* ≥ 0.05; *, *p* < 0.05; **, *p* < 0.01; ***, *p* < 0.001; and ****, *p* < 0.0001.

## 3 RESULTS

### 3.1 Identification of shared target genes between iron metabolism disorder and OA

**FIGURE 1:**
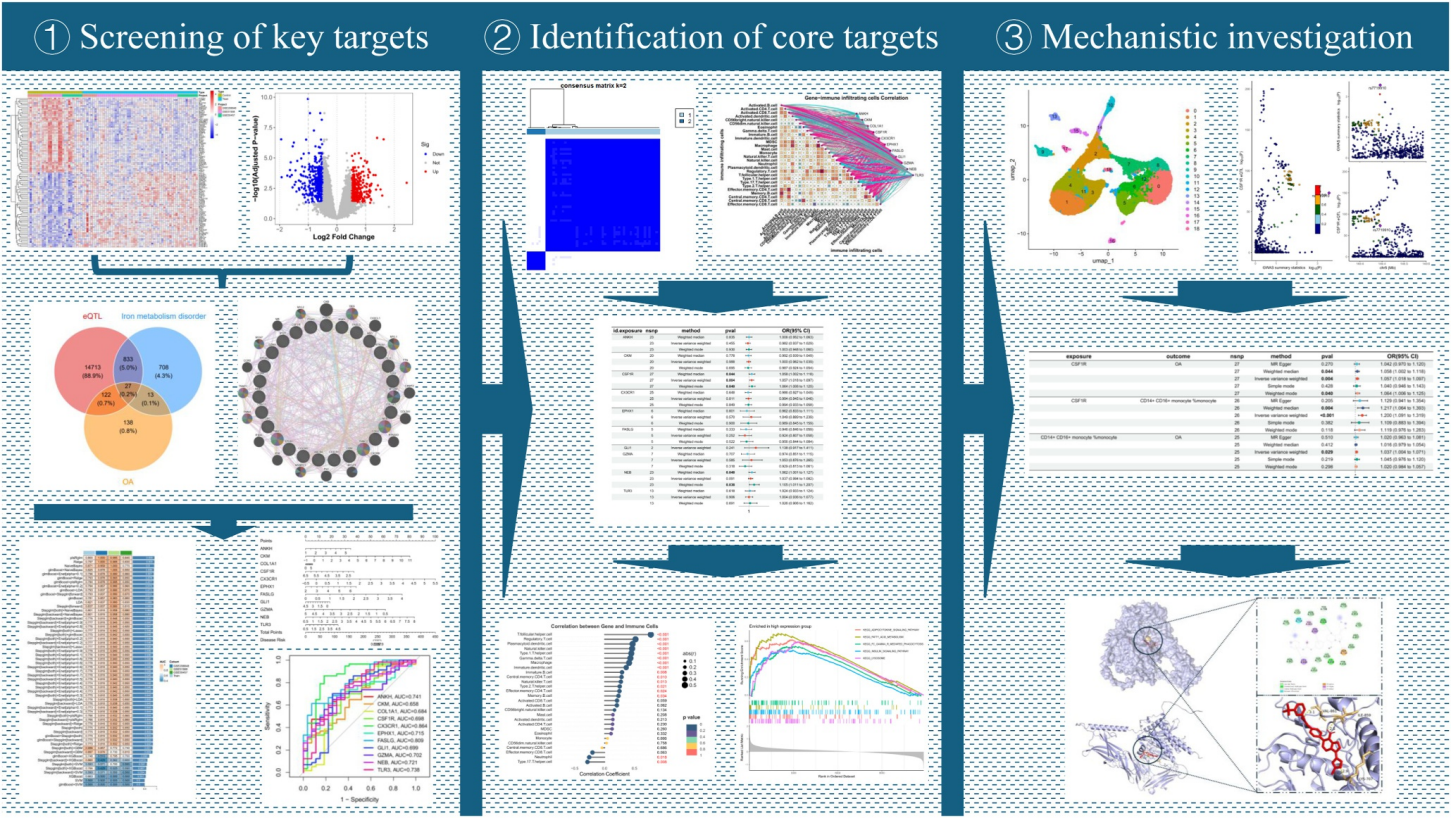
Workflow diagram.

**FIGURE 2:**
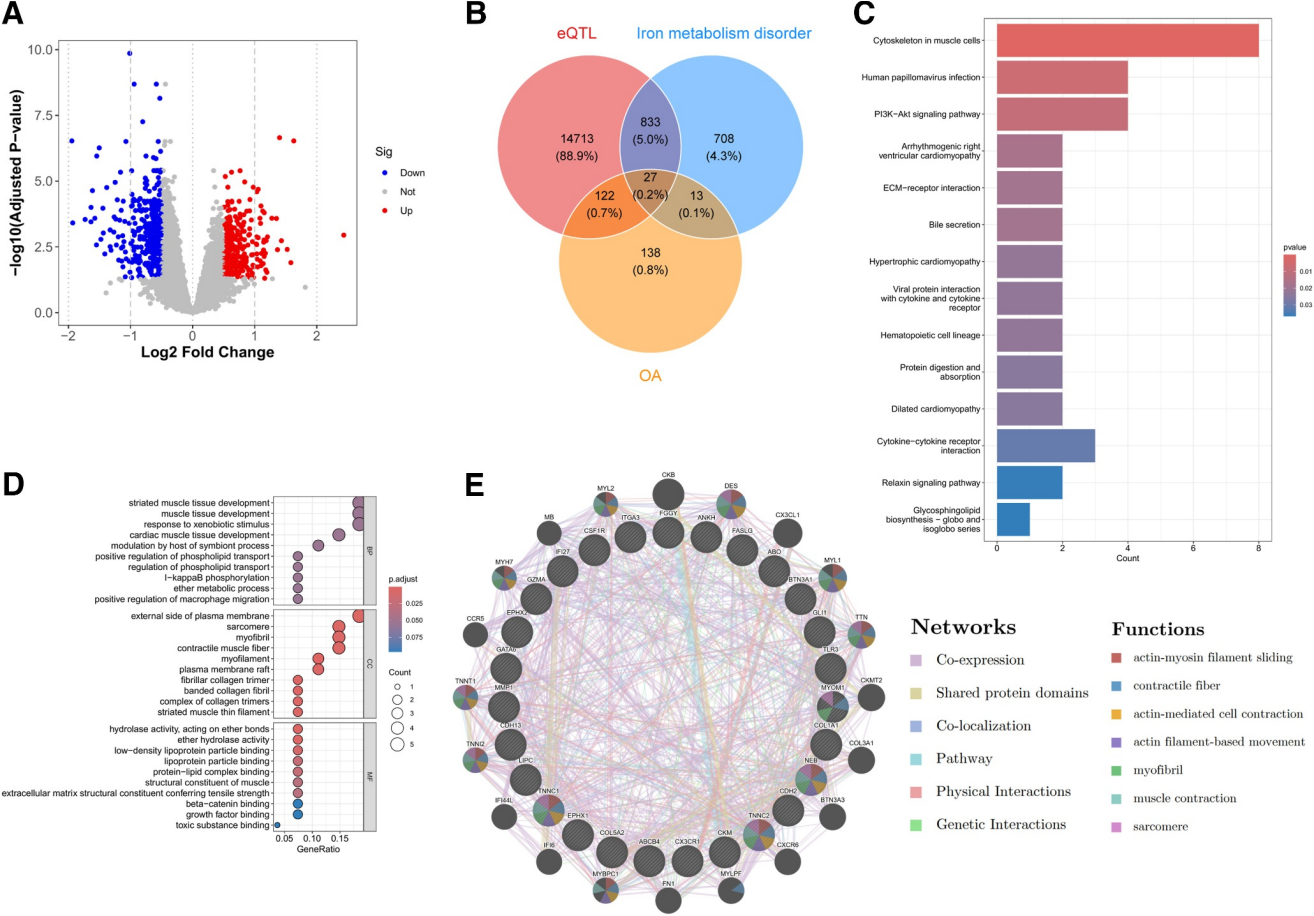
(A) Volcano plot of differentially expressed genes in osteoarthritis; (B) Venn diagram showing the overlap among genes associated with iron metabolism disorder, osteoarthritis‐related DEGs, and genes with expression quantitative trait loci data; (C) KEGG pathway enrichment analysis of the intersecting genes; (D) GO enrichment analysis of the intersecting genes; (E) PPI network of the intersecting genes. DEGs, differentially expressed genes; GO, gene ontology; KEGG, Kyoto encyclopedia of genes and genomes; PPI, protein–protein interaction.

The overall technical roadmap of this study is shown in Figure 1. MR analysis revealed a significant positive association between iron metabolism disorder and the risk of OA (Figure S1a–c), indicating a potential causal contribution of iron dysregulation to disease development. Based on three OA‐related transcriptomic datasets from the GEO database, batch effect correction and differential expression analysis identified a total of 642 DEGs, including 300 upregulated and 342 downregulated genes (Figure S2a–c; Figure 2A).

A total of 1581 iron metabolism‐related genes were retrieved from the GeneCards database. By intersecting iron metabolism‐related genes, OA‐associated DEGs, and genes with expression quantitative trait loci (eQTL) information, 27 shared candidate target genes were identified as potential mediators linking iron metabolism dysregulation to OA (Figure 2B).

### 3.2 Functional enrichment and PPI network analysis of shared genes

To further characterize the biological roles of the shared genes, GO and KEGG enrichment analyses were performed. GO analysis demonstrated that these genes were primarily enriched in biological processes such as muscle tissue development, regulation of phospholipid transport, IκB phosphorylation, positive regulation of macrophage migration, and myofibril assembly. In terms of molecular functions, enrichment was observed in protein–lipid complex binding and β‐catenin binding (Figure 2D). KEGG pathway analysis revealed significant enrichment in pathways related to cytoskeletal regulation, PI3K–Akt signaling, extracellular matrix–receptor interaction, protein digestion and absorption, cytokine–receptor interaction, and relaxin signaling (Figure 2C).

Furthermore, the PPI network constructed using GeneMANIA indicated extensive functional connectivity and physical interactions among the 27 shared genes, forming a tightly interconnected regulatory network (Figure 2E).

### 3.3 Construction of predictive models and identification of key targets

**FIGURE 3:**
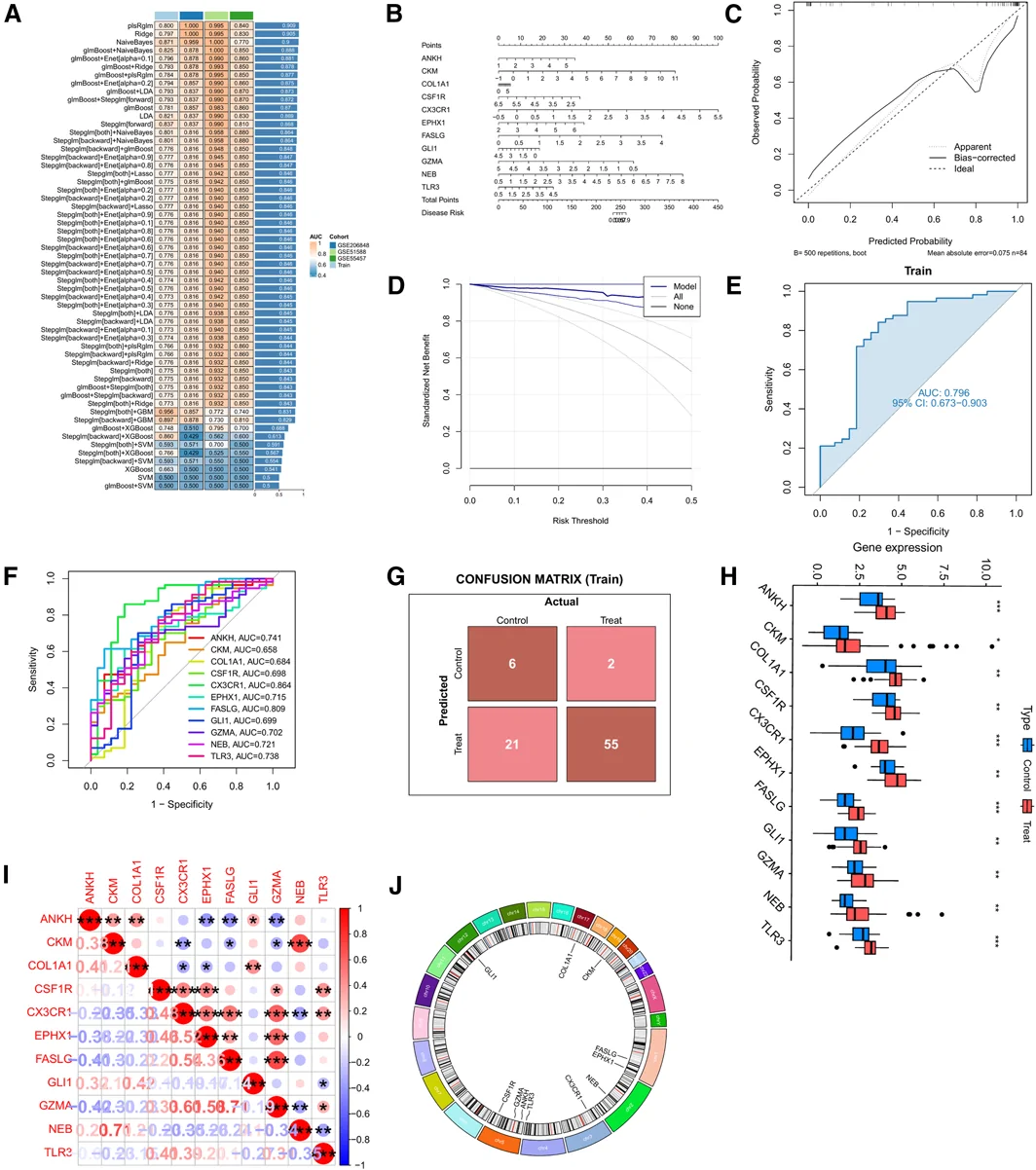
(A) Predictive models constructed using 12 machine learning algorithms; (B) nomogram for predicting osteoarthritis risk; (C) calibration curve of the nomogram; (D) DCA; (E) ROC curve of the training cohort; (F) ROC curve based on 11 key genes; (G) confusion matrix for the training cohort; (H) expression levels of the 11 key genes in healthy control and osteoarthritis groups. In this figure, “Control” represents healthy control samples without osteoarthritis, whereas “Treat” represents OA samples; (I) correlation matrix of the 11 key genes; (J) chromosomal locations of the key genes. DCA, decision curve analysis; OA, osteoarthritis; ROC, receiver operating characteristic.

A total of 113 predictive models were developed by integrating 12 machine learning algorithms to evaluate OA risk associated with iron metabolism dysregulation. Model performance was assessed in both training and testing cohorts, and the glmBoost combined with elastic net (*α* = 0.1) model demonstrated the optimal predictive performance (Figure 3A). Based on this model, 11 key genes were identified, including *ANKH, CKM, COL1A1, CSF1R, CX3CR1, EPHX1, FASLG, GLI1, GZMA, NEB,* and *TLR3*. To further evaluate model robustness and clinical utility, a nomogram was constructed, and its performance was assessed using calibration curves and DCA, which indicated favorable predictive capability and potential clinical applicability (Figure 3B–D). ROC analysis further confirmed the discriminative power of both the predictive model and the selected key genes, demonstrating high accuracy (Figure S2d–f; Figure 3E,F). In addition, confusion matrix analysis showed improved classification performance in both training and validation cohorts (Figure S2i; Figure 3G).

### 3.4 Characterization of key genes

Correlation analysis showed that most of the 11 key genes exhibited positive correlations with each other (Figure 3I). In addition, the expression levels of these genes were higher in OA samples compared with Control samples (Figure 3H).

Chromosomal mapping indicated that these key genes were distributed across multiple chromosomes, including chromosome 5 (*ANKH, CSF1R,* and *GZMA*), chromosome 19 (*CKM*), chromosome 17 (*COL1A1*), chromosome 3 (*CX3CR1*), chromosome 1 (*EPHX1* and *FASLG*), chromosome 12 (*GLI1*), chromosome 2 (*NEB*), and chromosome 4 (*TLR3*) (Figure 3J,K).

### 3.5 Subtype classification

**FIGURE 4:**
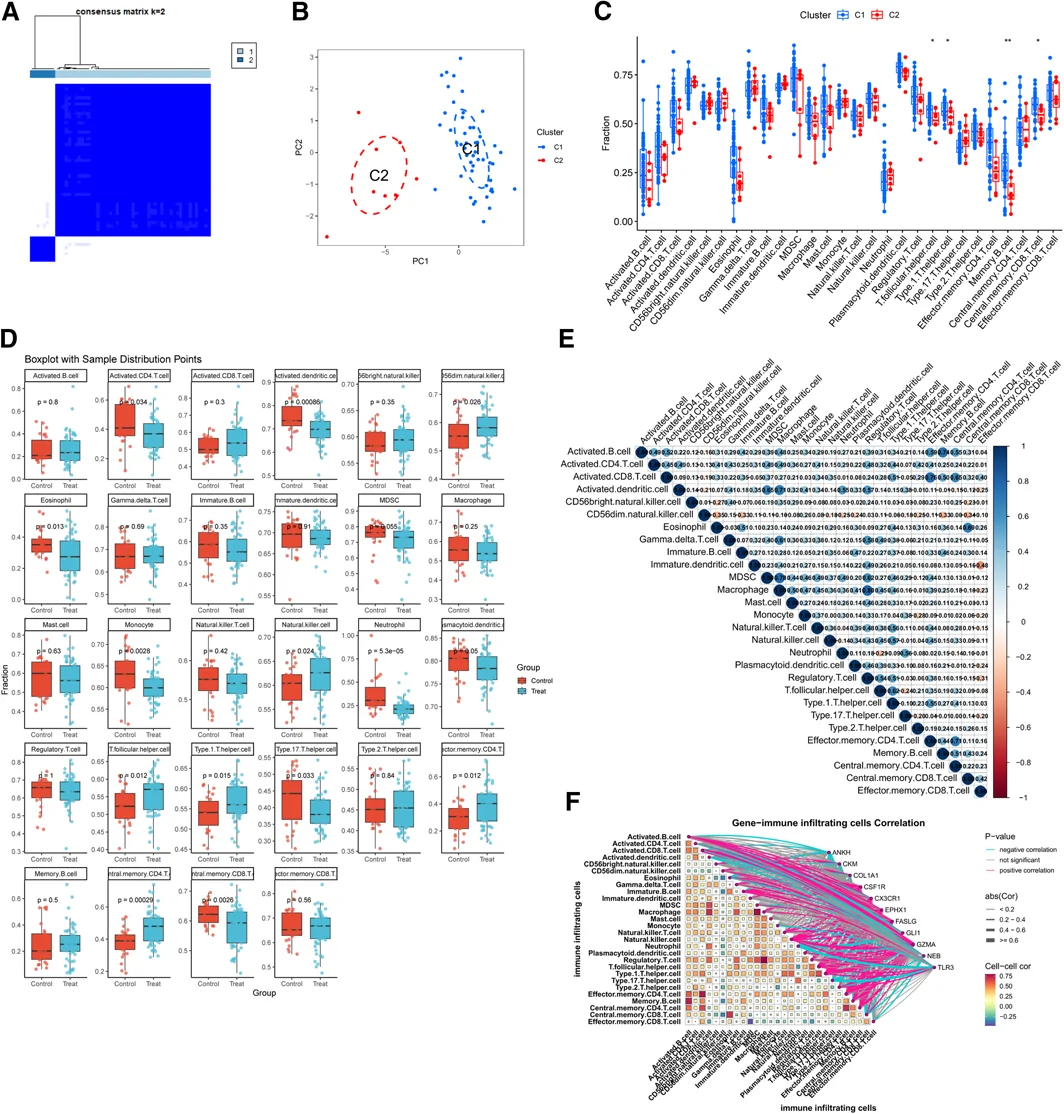
(A) Consensus clustering matrix when *k* = 2; (B) principal component analysis validation of the two consensus clusters; (C) boxplot comparing the relative abundance of immune cells between the two clusters; (D) boxplot showing the infiltration levels of 28 types of immune cells in osteoarthritis versus healthy groups; (E) heatmap of correlations among the 28 immune cell types; (F) the heatmap of correlations between 11 key genes and immune cell types.

Based on the expression profiles of the 11 key genes, unsupervised clustering using the k‐means algorithm stratified OA samples into two distinct subtypes, including subtype 1 (*n* = 49) and subtype 2 (*n* = 8) (Figure 4A). Evaluation of clustering stability indicated that *k* = 2 provided the optimal partitioning of the dataset (Figure S3a,b).

Differential expression analysis between subtypes revealed that *CKM* and *NEB* were more highly expressed in subtype 2 (Figure S3c,d). PCA further demonstrated a clear separation between the two subtypes, indicating distinct transcriptional profiles (Figure 4B). Immune infiltration analysis showed notable differences in the immune microenvironment between subtypes (Figure 4C). Specifically, subtype 1 exhibited higher proportions of T follicular helper cells, type 1 T helper (Th1) cells, memory B cells, and central memory CD8^+^ T cells.

### 3.6 Immune cell infiltration analysis

To further elucidate the infiltration characteristics of immune cells in OA, the ssGSEA algorithm was used to analyze the expression of 28 immune cell types in OA samples. The results showed statistically significant differences in the expression of 14 immune cell types between the disease and healthy groups, including activated CD4^+^ T cells, CD56bright natural killer cells, eosinophils, gamma delta T cells, natural killer T cells, neutrophils, regulatory T cells, T follicular helper cells, Type 17 T helper cells (Th17), Type 2 T helper cells (Th2), effector memory CD4^+^ T cells, central memory B cells, effector memory CD8^+^ T cells, and central memory CD8^+^ T cells (Figure 4D). Moreover, most of these immune cells were positively correlated with each other (Figure 4E).

Correlation analysis between the 11 key genes and immune cell infiltration revealed a positive correlation between the expression of key genes and the infiltration of several immune cell types (Figure 4F). This suggests that immune cell infiltration plays a crucial role in the pathogenesis and progression of the disease.

### 3.7 MR analysis

**FIGURE 5:**
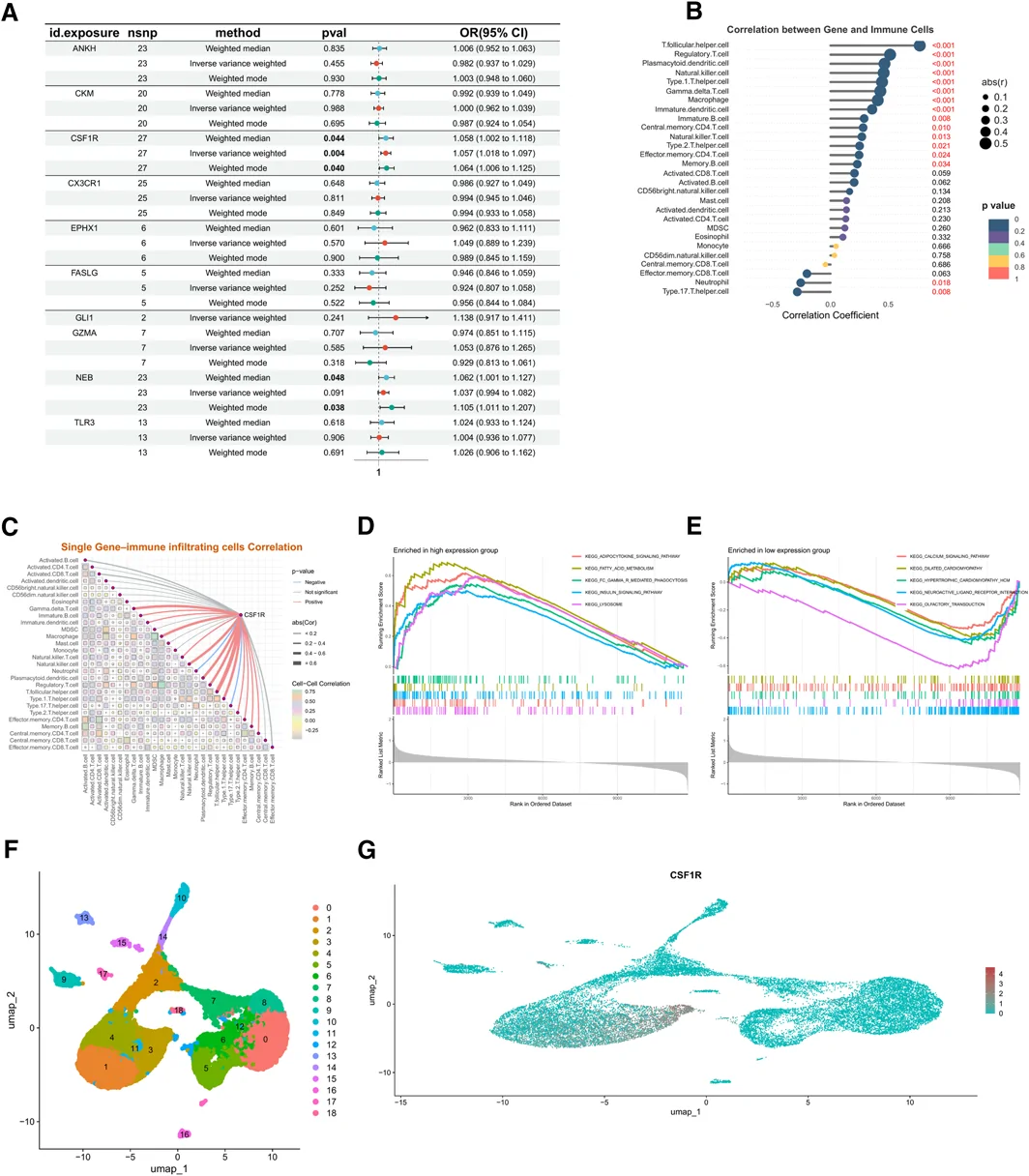
(A) Forest plot of Mendelian randomization analysis between key genes and osteoarthritis; (B, C) correlation between the core gene *CSF1R* and immune cell infiltration; (D, E) GSEA results of the core gene *CSF1R*; (F) UMAP plot showing clusters represented by colored dots, each labeled with group identifiers, possibly indicating distinct cell types or samples; (G) UMAP plot displaying expression patterns of the core gene. The figure shows gene expression across various cell types, with different clusters color‐coded to illustrate the spatial expression distribution of the gene. GSEA, gene set enrichment analysis; UMAP, uniform manifold approximation and projection.

To further validate the causal relationship between the 11 key genes and OA, MR analysis was performed. Because only one SNP data point was available for *COL1A1*, it could not be included in the MR analysis. The results indicated that *CSF1R* was positively associated with the progression of OA (OR = 1.057, *p* < 0.05), whereas the associations of the other 9 key genes were not statistically significant (Figure 5A). Thus, *CSF1R* was identified as the core target for further analysis.

### 3.8 Correlation analysis of core gene and immune cell infiltration

Correlation analysis was performed to explore the relationship between *CSF1R* and immune cell infiltration. The results showed that the expression of the core gene *CSF1R* was positively correlated with T follicular helper cells, regulatory T cells, plasmacytoid dendritic cells, natural killer cells, Type 1 T helper cells, gamma delta T cells, macrophages, immature dendritic cells, immature B cells, central memory CD4 T cells, natural killer T cells, Type 2 T helper cells, effector memory CD4 T cells, and memory B cells. However, it was negatively correlated with neutrophils and Type 17 T helper cells (Figure 5B,C, Figure S3e–t). These findings suggest that the core gene may influence the progression of OA by regulating immune cell infiltration.

### 3.9 GSEA and single‐cell analysis of core genes

GSEA analysis was performed to further investigate the roles of the core genes in biological processes, signaling pathways, and cellular functions. The results showed that the core genes were primarily enriched in the adipocytokine signaling pathway, fatty acid metabolism pathway, Fcγ receptor‐mediated phagocytosis pathway, insulin signaling pathway, and lysosome‐related pathways (Figure 5D,E).

**FIGURE 6:**
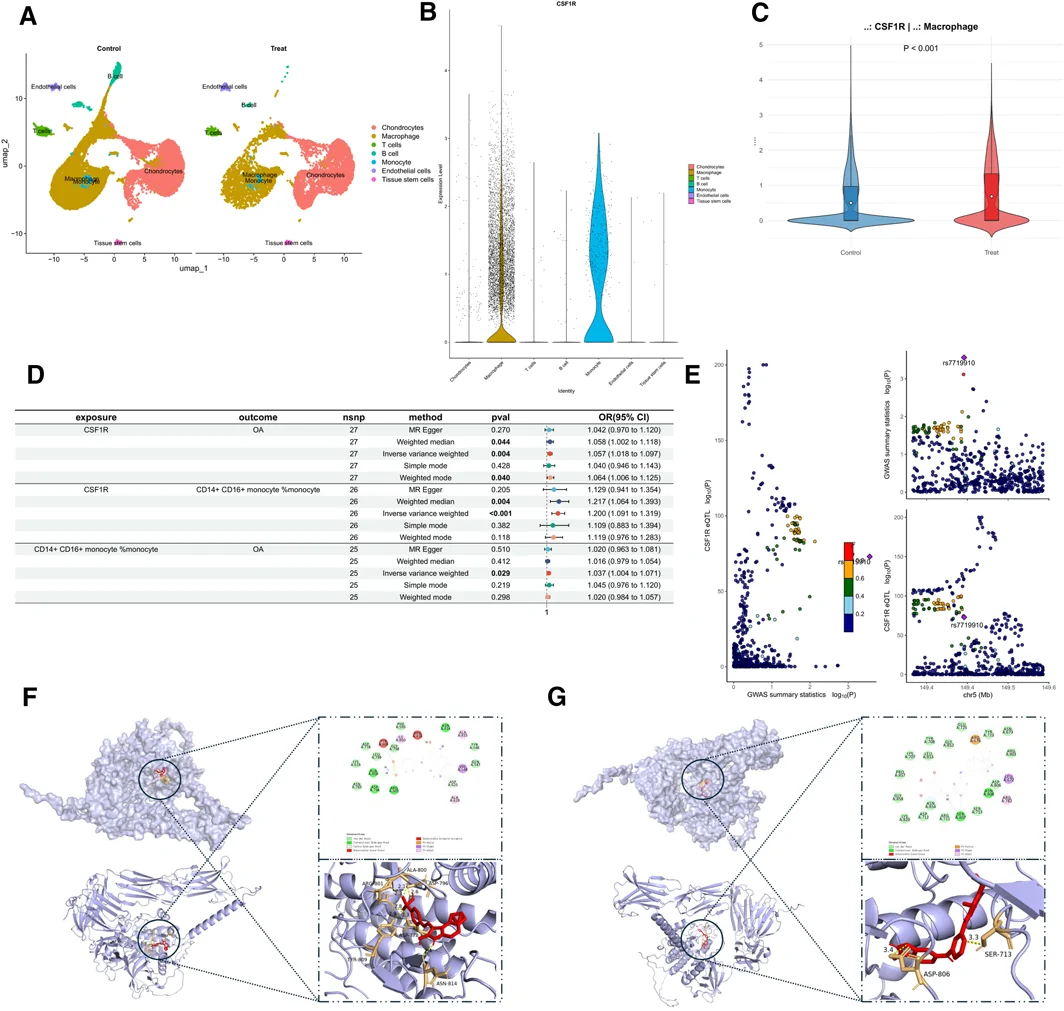
(A) UMAP plot with data points colored by cell types, such as chondrocytes, macrophages, and T cells, visualizing the distribution of cell populations based on gene expression; (B) violin plot showing the expression of *CSF1R* across chondrocytes, macrophages, and T cells. The width of the plot reflects data density at different expression levels, allowing for comparison of expression distributions; (C) violin plot of *CSF1R* expression specifically in macrophages; (D) forest plot of mediation analysis involving the core gene *CSF1R*, immune cell subpopulations, and osteoarthritis; (E) genomic locus map showing colocalization analysis of the core gene CSF1R with osteoarthritis‐associated regions; (F) molecular docking diagram illustrating the interaction between *CSF1R* and GW589933X; (G) molecular docking diagram illustrating the interaction between *CSF1R* and GW612286X. UMAP, uniform manifold approximation and projection.

To explore the cellular expression distribution of the core gene, single‐cell analysis was conducted. In OA‐related single‐cell transcriptomic data, after linear dimensionality reduction, UMAP clustering categorized the cells into 18 distinct cell types (Figure 5F). Single‐cell RNA sequencing analysis demonstrated that CSF1R expression displayed a cell‐type‐specific distribution pattern within the OA joint microenvironment. CSF1R expression was predominantly detected in macrophage and monocyte populations, whereas relatively limited expression was observed in other identified cell clusters (Figures 5G and 6A–C). These results suggest that the core gene may be involved in metabolic regulation and immune cell function.

### 3.10 Mediation analysis

Based on the previous analysis, the core gene *CSF1R* was found to be significantly associated with metabolism and immune cells. Therefore, this study further conducted a mediation analysis to explore the mediating role of metabolites and immune cells in the core gene's promotion of OA progression. First, MR analysis was performed to evaluate the causal associations of 1400 metabolites and 731 immune cell types with OA. The analysis revealed that 15 immune cell types were positively associated with OA progression (Supporting Information S1: Table S4), and 10 metabolites were positively associated with OA progression (Supporting Information S1: Table S5). Next, MR analysis was used to assess the association between the core gene and these 15 immune cell types and 10 metabolites (Supporting Information S1: Tables S6 and S7). Finally, the mediation analysis showed that the association between the 10 metabolites positively correlated with OA and the core gene was not statistically significant. However, CD14^+^ CD16^+^ monocyte %monocyte exhibited a significant positive mediating effect between *CSF1R* and OA, suggesting that *CSF1R* may promote OA progression in hereditary hemochromatosis by regulating this specific cell subset (Figure 6D).

### 3.11 Co‐localization analysis and molecular docking

**FIGURE 7:**
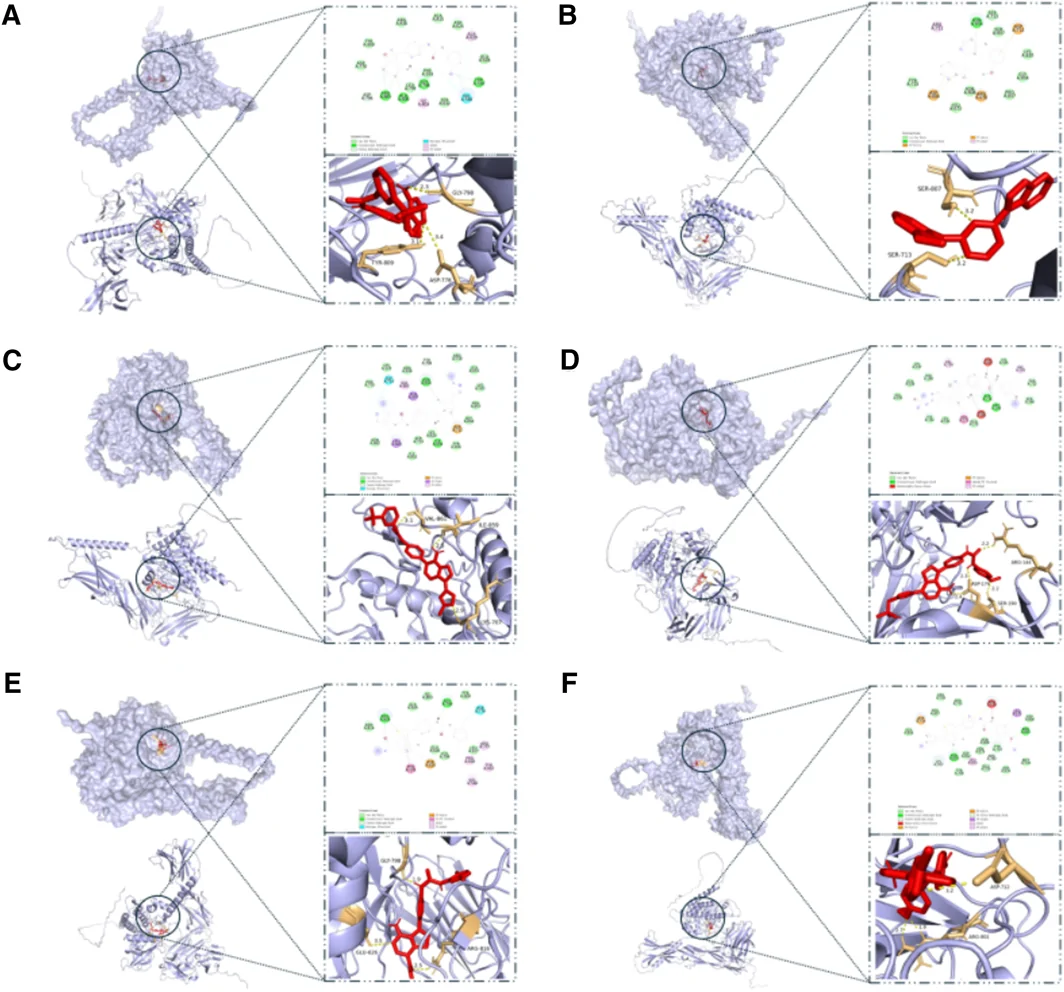
Molecular docking analysis of candidate compounds with the core gene CSF1R: (A) GW795493X; (B) GW810576X; (C) Kinome_439; (D) Kinome_450; (E) Kinome_474; (F) Kinome_490.

**FIGURE 8:**
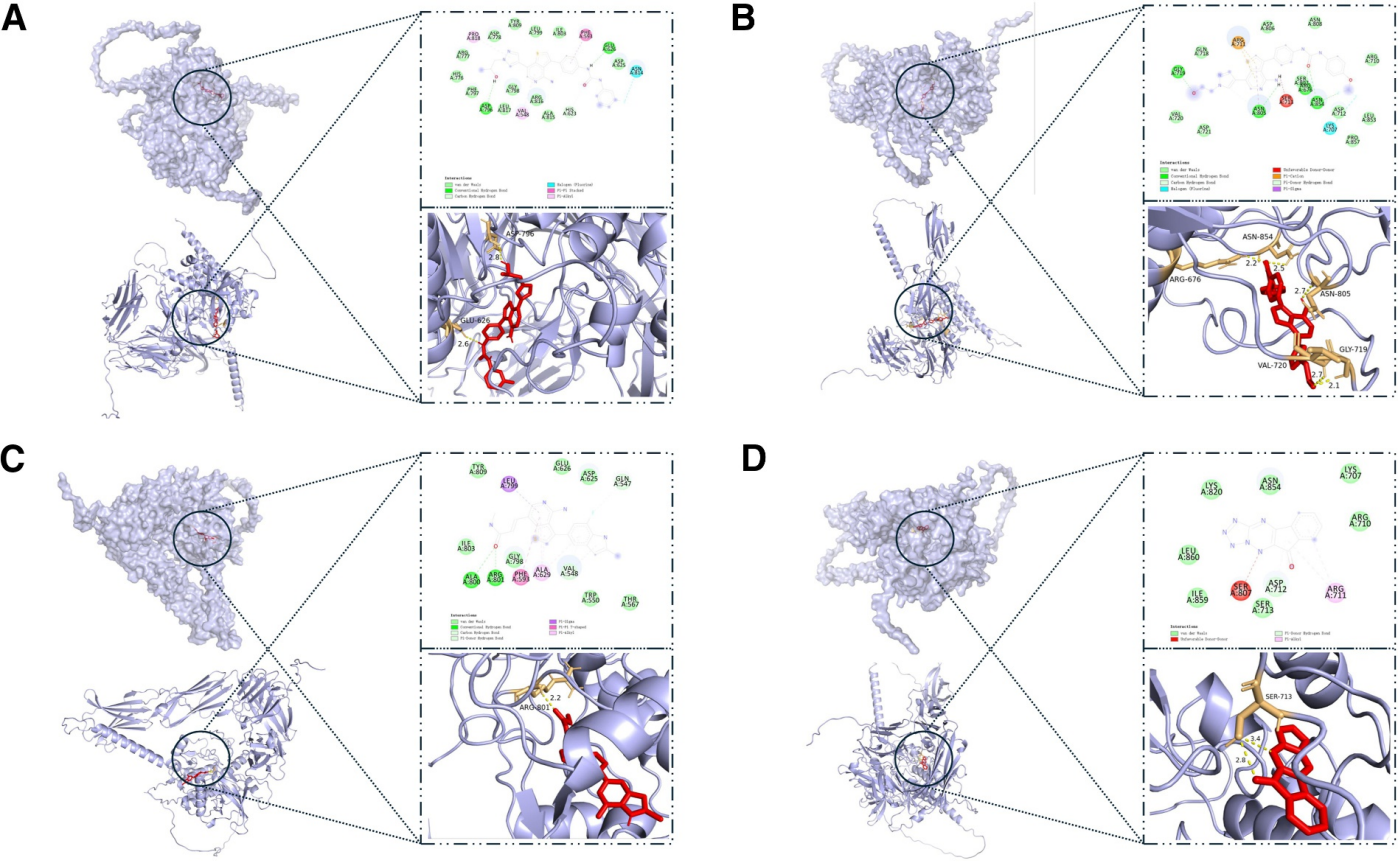
Molecular docking analysis of additional candidate compounds with the core gene CSF1R: (A) Kinome_504; (B) Kinome_506; (C) Kinome_2812; (D) PDK1/Akt/Flt dual pathway inhibitor.

Co‐localization analysis revealed a strong association between the core gene *CSF1R* and OA, with rs7719910 identified as a shared genetic regulatory locus between the two (Figure 6E). Subsequently, potential therapeutic drugs targeting *CSF1R* were screened from the DSigDB database, resulting in 13 candidate drugs (Supporting Information S1: Table S8). Molecular docking analysis was performed to evaluate the predicted binding interactions between candidate compounds and *CSF1R*. Compounds with docking energies lower than −5.0 kcal/mol were selected for further evaluation based on their relatively stronger predicted binding affinity. Compounds with docking energies higher than −5.0 kcal/mol were excluded from downstream analyses due to insufficient predicted interaction strength. The results indicated that Urea, N‐[4‐[7‐amino‐3‐(1‐methyl‐1H‐pyrazol‐4‐yl)pyrazolo[1,5‐a]pyrimidin‐6‐yl]phenyl]‐N'‐[3‐(trifluoromethyl)phenyl]‐ had the lowest binding energy (Supporting Information S1: Table S8). On the other hand, the binding energy of 1‐[4‐(4‐Aminofuro[2,3‐d]pyrimidin‐5‐yl)phenyl]‐3‐[2‐fluoro‐5‐(trifluoromethyl)phenyl]urea; hydrochloride was below −5.0 kcal/mol, suggesting that this compound is not suitable as a potential therapeutic drug. Finally, molecular binding sites of the 12 drugs with the target were predicted (Figures 6, 7, and 8A–D).

### 3.12 MD simulation

**FIGURE 9:**
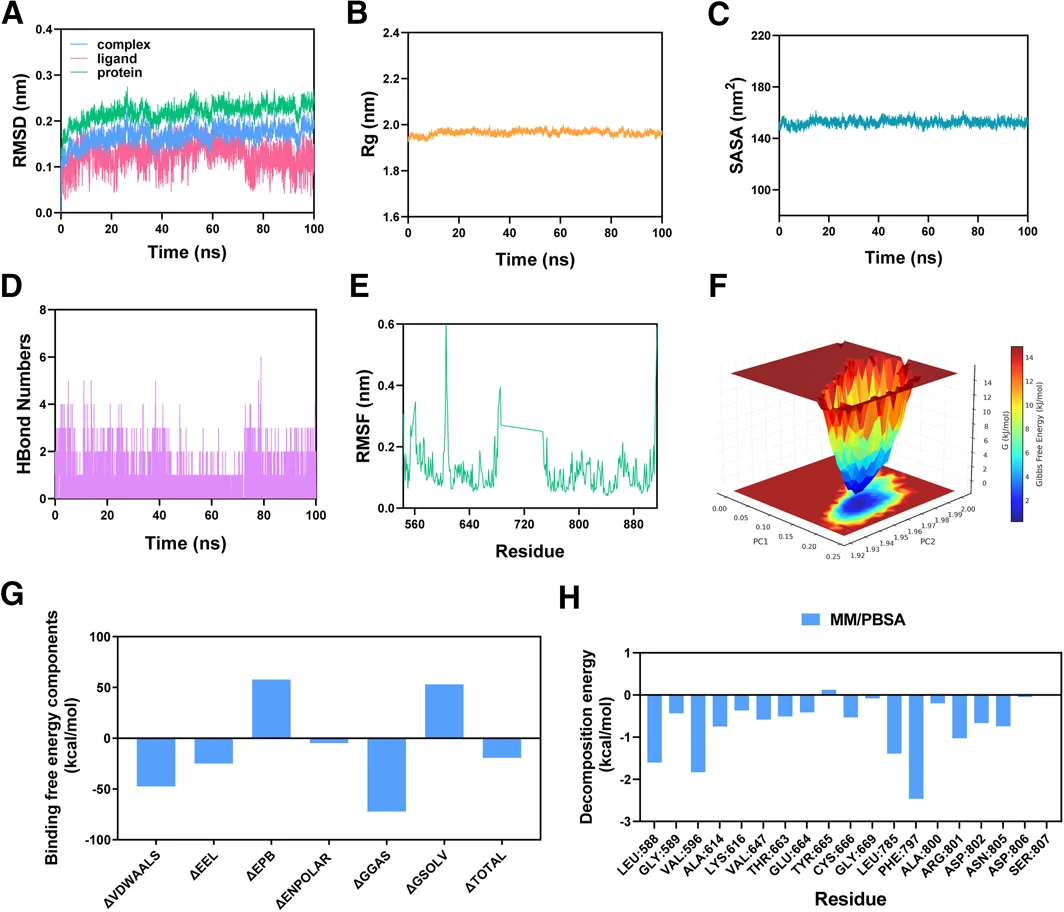
Molecular dynamics simulation of the ligand–target protein complex. (A) RMSD analysis of the apo protein, ligand, and protein–ligand complex during 100 ns simulation. (B) Rg analysis of the protein–ligand complex. (C) SASA analysis. (D) Hydrogen bond interactions between the ligand and target protein. (E) RMSF analysis of protein residues. (F) FEL analysis of the protein–ligand complex. (G) Per‐residue energy decomposition analysis of ligand–protein interactions. (H) Binding free energy calculation using MM/PBSA. FEL, free energy landscape; Rg, radius of gyration; RMSD, root mean square deviation; RMSF, root mean square fluctuation; SASA, solvent‐accessible surface area.

Molecular docking analysis identified Kinome_439 as the top‐ranked candidate with the lowest binding energy toward the core target, suggesting its potential as a therapeutic compound for iron metabolism‐associated OA. To investigate whether the ligand association could maintain a stable interaction with the target protein, a series of MD analyses were performed. The structural evolution of different simulation systems was initially examined by calculating the RMSD throughout the simulation trajectory. As illustrated in Figure 9A, the backbone deviations of both the free protein and ligand‐bound complex gradually stabilized after approximately 40 ns, with average RMSD values maintained around 0.24 and 0.20 nm, respectively. In comparison, the isolated ligand required a longer equilibration period and reached a stable state after approximately 75 ns, with fluctuations restricted to approximately 0.15 nm. The limited conformational drift observed in the complex indicates that ligand accommodation within the binding pocket does not induce substantial rearrangement of the target protein structure. The global compactness of the protein–ligand complex was further monitored using the radius of gyration (Rg). Throughout the 100 ns simulation period, the Rg profile exhibited only minor variations (Figure 9B), suggesting that the overall folding architecture of the protein remained preserved after ligand binding. No apparent expansion or collapse of the molecular structure was detected, indicating that the complex maintained a stable three‐dimensional organization under dynamic conditions. Changes in protein surface exposure were assessed by analyzing solvent‐accessible surface area (SASA). As shown in Figure 9C, the SASA trajectory of the ligand‐bound protein remained relatively constant during the simulation. The absence of pronounced SASA fluctuations suggests that ligand binding did not trigger significant conformational opening or structural exposure of buried regions, further demonstrating the stable accommodation of the ligand within the target binding site. Non‐covalent interactions, particularly hydrogen bonding, contribute substantially to ligand recognition and stabilization. The dynamic variation of hydrogen bonds formed between the ligand and target protein was analyzed (Figure 9D). During the simulation, the hydrogen bond number fluctuated between 0 and 6, with approximately two hydrogen bonds being maintained for the majority of the trajectory. This persistent interaction pattern indicates that polar contacts between the ligand and protein provide additional energetic support for complex stabilization. To characterize residue‐level flexibility, RMSF analysis was performed. As presented in Figure 9E, most amino acid residues displayed relatively restricted fluctuations, with RMSF values predominantly below 0.6 nm. Notably, the residues surrounding the ligand‐binding region exhibited limited mobility, suggesting that ligand engagement contributes to maintaining a relatively rigid interaction environment. The conformational energy distribution of the complex was subsequently explored using free energy landscape (FEL) analysis. The FEL map revealed distinct energy basins corresponding to different conformational states (Figure 9F). A predominant low‐energy region was observed around PC1 = 0.17 and PC2 = 1.955, representing the most favorable conformational ensemble sampled during the simulation. The enrichment of the complex within this low‐energy basin indicates that the ligand‐bound system preferentially adopts a stable equilibrium configuration. The energetic favorability of ligand binding was further quantified using the MM/PBSA approach. The calculated binding free energy of the complex was −19.18 kcal/mol (Figure 9H), reflecting a spontaneous and energetically favorable association between the ligand and target protein. Subsequent residue decomposition analysis identified PHE797, VAL596, LEU588, LEU785, ARG801, ASN805, ALA614, and ASP802 as major contributors to ligand stabilization (Figure 9G). These residues may constitute key interaction hotspots responsible for maintaining ligand recognition and binding affinity. Taken together, the MD simulation results demonstrate that the ligand–protein complex achieves a stable conformational state during dynamic evolution. The combination of favorable energetic contributions, sustained intermolecular interactions, and limited structural fluctuation provides molecular‐level evidence supporting the potential binding capability of the ligand toward the target protein. Collectively, these results demonstrate that the Kinome_439–target complex maintains structural stability and favorable binding interactions throughout the simulation.

### 3.13 Experimental validation

To validate the expression pattern of the core target under inflammatory conditions, WB analysis was performed. Compared with the control group, *CSF1R* protein expression was markedly elevated in the inflammatory model. This observation is consistent with the bioinformatics findings, supporting the involvement of *CSF1R* activation in the OA‐associated inflammatory microenvironment (Figure S4a,b).

### 3.14 Single‐cell perturbation simulation of *CSF1R*

**FIGURE 10:**
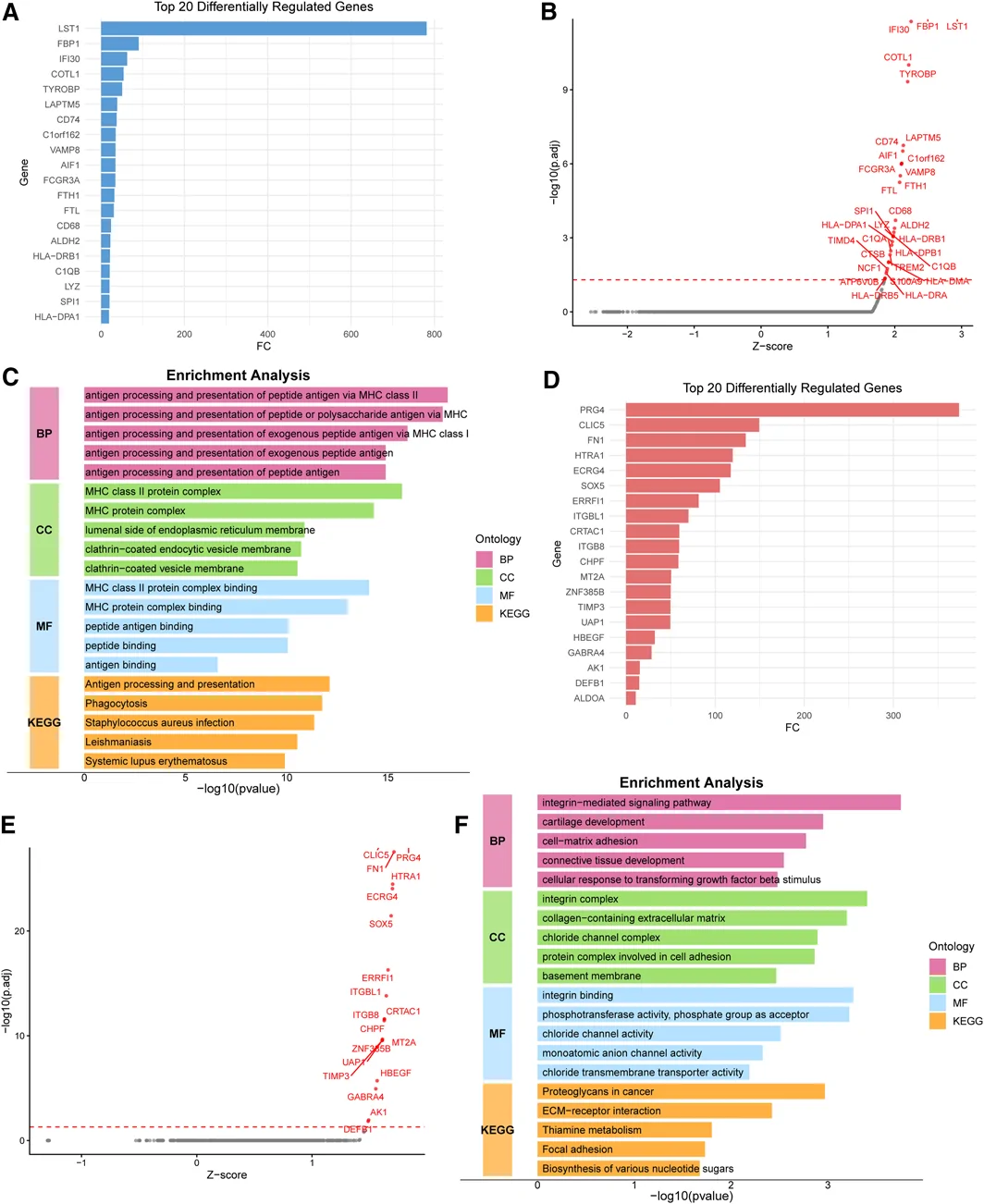
Single‐cell perturbation simulation reveals transcriptomic responses and functional network alterations following *CSF1R* perturbation. (A) Top 20 genes exhibiting the strongest transcriptional responses following simulated *CSF1R* knockout. (B) Genome‐wide distribution of perturbation‐responsive genes after *CSF1R* knockout. (C) Functional enrichment analysis of genes responsive to *CSF1R* loss‐of‐function, including GO, BP, CC, MF, and KEGG pathway analyses. (D) Top 20 genes showing the greatest transcriptional responses following simulated CSF1R overexpression. (E) Genome‐wide distribution of perturbation‐responsive genes after CSF1R overexpression. (F) GO and KEGG enrichment analyses of genes responsive to CSF1R gain‐of‐function perturbation. BP, biological process; CC, cellular component; GO, gene ontology; KEGG, Kyoto encyclopedia of genes and genomes; MF, molecular function.

To further characterize the regulatory role of *CSF1R* within the OA immune microenvironment, single‐cell gene perturbation simulations were performed by reconstructing gene regulatory networks and modeling both *CSF1R* loss‐of‐function and gain‐of‐function states. Following simulated *CSF1R* knockout, *LST1* exhibited the most prominent transcriptional response among all genes (Figure 10A,B). Functional enrichment analysis of perturbation‐responsive genes demonstrated significant enrichment of biological processes associated with major histocompatibility complex (MHC) class II‐mediated antigen processing and presentation. Consistently, enriched cellular components were predominantly related to MHC protein complexes, whereas molecular functions were characterized by MHC protein complex binding. KEGG pathway analysis further revealed significant enrichment in antigen processing and presentation, phagosome, and *Staphylococcus aureus* infection, highlighting a network shift toward antigen presentation and innate immune functions following *CSF1R* depletion (Figure 10C).

In contrast, simulated *CSF1R* overexpression identified *PRG4* as the most responsive downstream gene (Figure 10D,E). Enrichment analysis of the perturbed gene set indicated significant overrepresentation of biological processes involved in integrin‐mediated signaling and cartilage development. The corresponding cellular components were primarily associated with integrin complexes and the collagen‐containing extracellular matrix, whereas molecular functions were enriched for integrin binding and phosphotransferase activity with phosphate‐containing group acceptors. KEGG analysis demonstrated enrichment in proteoglycans in cancer, ECM–receptor interaction, and thiamine metabolism pathways (Figure 10F). Collectively, these perturbation simulations suggest that alterations in *CSF1R* activity are accompanied by coordinated regulatory changes involving immune‐related pathways as well as extracellular matrix remodeling and cartilage‐associated biological programs.

## 4 DISCUSSION

By integrating multi‐layered analytical approaches, this study provides a systematic characterization of the molecular mechanisms linking iron metabolism dysregulation to OA progression, and identifies *CSF1R* as a central regulatory node in this process. The findings delineate the cell type–specific expression pattern of *CSF1R* and its downstream regulatory landscape, thereby refining the current understanding of immunometabolic crosstalk in OA pathogenesis. Importantly, these results establish a mechanistic and translational framework supporting *CSF1R* as a potential therapeutic target, and suggest a multi‐level precision intervention strategy centered on *CSF1R* modulation, with implications for drug development and clinical management of OA.

It should be emphasized that the findings of this study specifically relate to knee OA rather than OA as a generalized disease entity. Knee OA exhibits unique pathological characteristics driven by the interaction between mechanical stress, inflammatory responses, and metabolic imbalance. Unlike certain OA phenotypes, such as hip OA, where structural degeneration may be more closely associated with developmental abnormalities or altered joint morphology, knee OA is strongly influenced by chronic weight‐bearing stress and progressive synovial immune activation. Macrophages and monocyte‐derived inflammatory populations have been increasingly recognized as key regulators of knee OA progression through modulation of inflammatory cytokine production, extracellular matrix degradation, and tissue remodeling.^19^, ^20^ Therefore, the identification of *CSF1R*‐dependent monocyte–macrophage communication in our study provides a mechanistic explanation that is particularly relevant to the inflammatory microenvironment of knee OA.

*CSF1R*, a key receptor tyrosine kinase governing the monocyte–macrophage lineage,^21^, ^22^, ^23^ emerges as a functionally relevant target in iron metabolism‐associated OA. Unlike conventional therapeutic strategies that primarily focus on symptomatic relief or cartilage preservation, *CSF1R* represents a critical molecular interface linking iron homeostasis imbalance with local immune‐inflammatory responses in joint tissues. *PRG4* (proteoglycan 4), also known as lubricin, is an essential component involved in joint lubrication and cartilage protection. Reduced *PRG4* expression has been associated with impaired joint surface integrity and increased susceptibility to cartilage degeneration.^24^, ^25^ Interestingly, our macrophage‐focused single‐cell perturbation analysis identified *PRG4* as a highly responsive downstream gene following *CSF1R* modulation, suggesting that immune cells may potentially participate in regulating joint‐protective pathways. Although the precise mechanism linking macrophage *CSF1R* signaling and *PRG4* regulation remains unclear, this finding provides a potential connection between immune regulation and tissue homeostasis in OA. The present findings support a model in which iron dysregulation is associated with aberrant activation of *CSF1R* signaling, promoting the expansion, infiltration, and activation of proinflammatory CD14^+^CD16^+^ monocyte subsets, thereby amplifying inflammatory cascades and accelerating cartilage matrix degradation. This proposed “iron overload–*CSF1R* activation–immune amplification–tissue degeneration” axis highlights a pathogenic loop that may underlie disease progression. Targeting *CSF1R* could therefore offer a mechanism‐oriented strategy to interrupt this cascade at an upstream level, rather than solely mitigating downstream clinical manifestations. Furthermore, the causal association between *CSF1R* and OA established through MR, together with the shared genetic signals identified by colocalization analysis, provides robust genetic evidence supporting the prioritization of *CSF1R* as a therapeutic target, thereby reducing the uncertainty commonly associated with target selection in drug development.

The integration of molecular docking and MD simulations provides structural insights into CSF1R‐targeted ligand interactions, thereby offering a mechanistic basis for the development of small‐molecule inhibitors. These analyses serve as a critical bridge linking computational discovery to pharmacological translation. Through screening of the DSigDB database, multiple candidate compounds targeting *CSF1R* were identified. Among them, Kinome_439 exhibited the lowest binding energy and stable interaction profiles with the *CSF1R* protein, with well‐defined binding sites. These findings highlight Kinome_439 as a potential lead compound and provide a structural framework for subsequent drug optimization. Rational modification of functional groups may further enhance binding affinity, specificity, and pharmacokinetic properties. In parallel with de novo drug development, drug repositioning strategies represent a feasible and efficient alternative. Given the emerging role of *CSF1R* inhibitors in inflammatory and immune‐related diseases, existing *CSF1R*‐targeted agents—particularly those currently undergoing clinical evaluation in oncology and autoimmune disorders—may be repurposed for the treatment of iron metabolism‐associated OA. Such an approach could substantially reduce development time and associated costs. Beyond small‐molecule inhibitors, monoclonal antibodies targeting *CSF1R* also represent a promising therapeutic modality. Owing to their high specificity, antibody‐based strategies may minimize off‐target effects commonly associated with kinase inhibitors and may be particularly suitable for long‐term intervention in chronic degenerative diseases such as OA. This approach may be especially relevant for early preventive strategies in populations at high risk of iron metabolism dysregulation.

Given the immunometabolic crosstalk underlying iron metabolism‐associated OA, therapeutic strategies centered on *CSF1R* targeting may benefit from combinatorial interventions. The present findings provide a mechanistic rationale for the development of multi‐layered treatment approaches aimed at improving therapeutic efficacy. One potential strategy involves the combination of *CSF1R* inhibitors with agents that modulate iron metabolism, such as iron chelators or hepcidin agonists. This approach may achieve a dual‐targeting effect by simultaneously correcting systemic or local iron overload and attenuating downstream *CSF1R*‐mediated inflammatory signaling, thereby exerting synergistic therapeutic effects. In addition, integrating systemic *CSF1R*‐targeted therapy with intra‐articular anti‐inflammatory treatments, including nonsteroidal anti‐inflammatory drugs or locally administered glucocorticoids, may further enhance therapeutic precision. Such a strategy could suppress local joint inflammation while minimizing systemic drug exposure and off‐target effects. Furthermore, combining *CSF1R*‐targeted intervention with strategies specifically targeting CD14^+^CD16^+^ monocyte subsets—such as selective depletion or functional inhibition—may provide an additional layer of disease control. This dual‐intervention approach has the potential to more effectively disrupt key pathogenic pathways and improve both specificity and overall therapeutic outcomes beyond single‐target modulation.

The machine learning‐based predictive model constructed in this study, incorporating *CSF1R* along with 11 key genes, provides a framework for the clinical translation of *CSF1R*‐targeted therapies. This model enables effective risk stratification of individuals with iron metabolism dysregulation, thereby facilitating early identification of populations at increased risk of OA. Such stratification may support the implementation of precision‐based and preventive therapeutic strategies. In high‐risk populations identified by the model, early intervention using low‐dose *CSF1R*‐targeted agents may represent a promising approach to delay or potentially mitigate disease onset and progression. This strategy aligns with the principles of precision medicine and early therapeutic intervention, with the potential to improve the overall benefit–risk profile of treatment. Furthermore, the panel of 11 key genes may serve as candidate biomarkers for companion diagnostics in *CSF1R*‐targeted therapy. Monitoring their expression patterns could enable the prediction of therapeutic responsiveness and facilitate the identification of patient subgroups most likely to benefit from treatment, thereby optimizing patient selection and reducing unnecessary interventions. Collectively, these findings establish *CSF1R* as a central pharmacological target in iron metabolism‐associated OA and provide mechanistic insights into its role in disease progression. The identification of *CSF1R*‐targeted lead compounds, together with the proposed combinatorial therapeutic strategies, outlines a translational framework for precision intervention. In addition, this work offers a conceptual basis for dual‐target pharmacological modulation of metabolic and immune pathways in degenerative joint diseases and may have broader implications for other chronic inflammatory conditions associated with metabolic dysregulation.

The enrichment of adipocytokine signaling and fatty acid metabolism pathways further suggests that *CSF1R*‐associated regulation may extend beyond local immune activation and involve broader metabolic communication within the joint microenvironment. Adipose tissue and articular cartilage are metabolically interconnected tissues, and adipokines released from adipose depots can influence chondrocyte metabolism, inflammatory signaling, and extracellular matrix homeostasis.^26^, ^27^ In the context of OA, dysregulated lipid metabolism and adipocytokine signaling may promote chronic inflammation and oxidative stress, thereby creating a microenvironment favorable for iron accumulation and iron‐dependent cellular damage.^28^, ^29^, ^30^ Macrophages represent a critical interface between metabolic regulation and iron homeostasis, as they participate in iron recycling, storage, and inflammatory responses.^31^ Therefore, alterations in fatty acid metabolism and adipocytokine‐related signaling may influence iron metabolism indirectly by reshaping macrophage polarization states, inflammatory activity, and ferroptosis susceptibility. Although our current computational analyses identify these pathways as potential biological connections, experimental validation using integrated adipose–cartilage models and iron perturbation systems will be required to establish direct mechanistic relationships.

Despite the comprehensive computational framework and multi‐dimensional analyses performed in this study, several limitations warrant consideration. First, the conclusions presented here are primarily based on bioinformatics analyses, molecular simulations, and computational predictions. Although these approaches provide valuable insights into the potential role of *CSF1R*‐mediated regulation and facilitate the identification of candidate therapeutic compounds, the pharmacological characteristics and biological efficacy of these compounds remain to be experimentally established. Future investigations should integrate cellular and animal validation systems, including models of iron metabolism imbalance and iron accumulation‐associated OA, to systematically assess therapeutic effects, dose‐dependent responses, temporal dynamics, and pharmacokinetic properties. Comprehensive evaluation of absorption, distribution, metabolism, and excretion characteristics will also be required to determine their translational feasibility. Second, the datasets analyzed in this study were obtained from publicly accessible repositories, and inherent differences among cohorts, including genetic background, disease severity, treatment history, and comorbidity profiles, may contribute to biological heterogeneity and potentially influence the external applicability of our findings. Therefore, validation in large‐scale multi‐center clinical cohorts will be essential to determine whether *CSF1R* expression patterns and associated molecular signatures can reliably predict therapeutic responses in patients with iron metabolism‐related knee OA. Although this study incorporated single‐cell transcriptomic data from synovial tissue and subchondral bone, two key immune‐regulatory compartments involved in OA progression, we acknowledge that articular cartilage represents an indispensable component of joint degeneration and repair processes. The lack of cartilage‐specific single‐cell resolution data represents an important limitation. Nevertheless, the primary objective of this study was to elucidate immune‐mediated regulatory mechanisms, and synovium and subchondral bone provide the most informative cellular niches for investigating macrophage and monocyte‐associated alterations. Future integration of cartilage, synovium, and subchondral bone through spatial transcriptomics, spatial proteomics, or multi‐compartment single‐cell sequencing approaches may enable the construction of a comprehensive molecular atlas of the OA joint. Third, although downstream signaling pathways associated with *CSF1R* activation were systematically characterized, the upstream regulatory mechanisms controlling *CSF1R* expression under iron dysregulation conditions remain incompletely understood. Further studies focusing on transcriptional regulation, chromatin remodeling, and epigenetic modifications during iron accumulation may uncover additional regulatory nodes and facilitate the development of more effective multi‐target intervention strategies. Another limitation concerns the experimental validation strategy, as mechanistic experiments were conducted using the murine macrophage cell line RAW264.7 rather than human‐derived macrophage systems. Although RAW264.7 cells are extensively utilized for studying macrophage activation and inflammatory responses, species‐specific differences in transcriptional regulation, signaling networks, and immune responses may limit direct extrapolation to human disease. Future validation using human primary macrophages, synovial macrophages isolated from OA patients, or patient‐derived tissue samples will be necessary to further establish the clinical relevance of *CSF1R*‐mediated regulation in human knee OA. Finally, the *CSF1R*‐targeting compounds identified through computational screening require further evaluation regarding their safety, toxicity, and drug‐development potential. Comprehensive preclinical assessments, including acute and chronic toxicity studies, off‐target profiling, and structural optimization to improve selectivity, stability, and bioavailability, will be critical before clinical translation. Collectively, addressing these limitations will provide essential evidence for advancing *CSF1R*‐targeted therapeutic strategies from computational discovery toward clinical application in OA.

## 5 CONCLUSION

This study provides an integrative analysis of the molecular mechanisms by which iron metabolism dysregulation contributes to OA progression, combining MR, single‐cell transcriptomics, machine learning, and mediation analysis within a unified framework. A total of 27 shared candidate genes linking iron metabolism and OA were identified, from which 11 key genes were further selected through predictive modeling. Among these, *CSF1R* was established as a core regulatory target with a causal association to OA. Multi‐level evidence demonstrated that *CSF1R* is highly expressed in macrophages and may mediate disease progression through CD14^+^CD16^+^ monocyte subsets, supported by pathway enrichment and genetic colocalization analyses. Collectively, these findings highlight *CSF1R* as a central molecular node connecting iron metabolism imbalance with immune regulation in OA, providing a mechanistic basis for targeted therapeutic strategies. Although certain limitations remain, including the need for experimental validation and potential dataset heterogeneity, this work offers a foundation for future studies on immunometabolic regulation and target‐based intervention in OA.

## AUTHOR CONTRIBUTIONS

Yue Zhou and Guohang Shen conceived and designed the study, performed the bioinformatics analyses, and drafted the manuscript. Lijing Si and Ruoyan Wang contributed to data collection, preprocessing, differential expression analysis, and visualization. Kaiyong Wang and Yang Chen performed machine learning modeling, Mendelian randomization, immune infiltration, mediation analysis, and single‐cell analysis. Yupei Dai supervised the study, provided methodological guidance, revised the manuscript critically, and approved the final version. All authors reviewed and approved the final manuscript.

## CONFLICT OF INTEREST STATEMENT

The authors declare no conflicts of interest.

## ETHICS STATEMENT

Ethical approval is not applicable to our study.

## Supporting information

Supporting Information S1

Supporting Information S2

Figure S1

Figure S2

Figure S3

Figure S4

## Data Availability

The data will be available from the corresponding author upon reasonable request.
