# Supplementary material for: Virtual single‐cell perturbation and genetic causal inference reveal CSF1R‐dependent immunometabolic communication in iron metabolism‐associated osteoarthritis
Source: J Cell Commun Signal. 2026 Aug 27;20(3):e70107. doi: 10.1002/ccs3.70107 (PMC13519958; doi:10.1002/ccs3.70107)
Supplement: Supplementary file 1 [file CCS3-20-e70107-s003.docx]

**Supplementary Table 1:** Osteoarthritis datasets obtained from the GEO database.

| ID | GEO dataset | Samples | Tissue source |
| --- | --- | --- | --- |
| 1 | GSE51588 | 40 OA patients and 10 normal controls | Subchondral bone |
| 2 | GSE55457 | 10 OA patients and 10 normal controls | Synovium |
| 3 | GSE206848 | 7 OA patients and 7 normal controls | Synovium |
| 4 | GSE248453 | 4 control groups, 4 observation groups | Synovium |

**Supplementary Table 2:** Database URLs.

| PubChem | <https://pubchem.ncbi.nlm.nih.gov/> |
| --- | --- |
| ChEMBL | <https://www.ebi.ac.uk/chembl/> |
| Uniprot | https://www.uniprot.org/ |
| GEO | [https://www.ncbi.nlm.nih.gov/geo/](https://www.ncbi.nlm.nih.gov/geo/%EF%BC%89) |
| RCSB PDB | [https://www.rcsb.org/](https://www.rcsb.org/%EF%BC%89) |
| GWAS | https://gwas.mrcieu.ac.uk/ |
| eQTLGen | https://www.eqtlgen.org/cis-eqtls.html |

**Supplementary Table 3:** Specific parameters set for machine learning algorithms.

The raw data was initially preprocessed by removing missing values and outliers. To standardize the features, Z-score normalization was applied, adjusting the mean of each feature to 0 and its standard deviation to 1, which helped eliminate the influence of different scales across features. The datasets (GSE51588, GSE55457, and GSE206848) were then randomly split into a training set (70%) and a test set (30%). During model training, we assessed the performance of several machine learning algorithms, such as Elastic Net Regression (λ=0.1), Lasso Regression (λ=0.05), Ridge Regression (λ=1.0), Support Vector Machine (SVM; C=1.0, γ=0.01), Linear Discriminant Analysis (LDA), Gradient Boosting Machine (GBM; learning rate=0.1, number of trees=100), Random Forest (RF; number of trees=200), and XGBoost (XGB; learning rate=0.01, number of trees=150). These models were trained on the training set, with hyperparameter optimization performed via cross-validation.

**Supplementary Table 4.** Mendelian randomization analysis results between immune cells and osteoarthritis.

| **id.exposure** | **id.outcome** | **outcome** | **exposure** | **method** | **nsnp** | **b** | **se** | **pval** | **lo_ci** | **up_ci** | **or** | **or_lci95** | **or_uci95** | **effect** |
| --- | --- | --- | --- | --- | --- | --- | --- | --- | --- | --- | --- | --- | --- | --- |
| GCST90001500 | z79XSR | 30664745-GCST007090-EFO_0004616.h.tsv | Activated & resting Treg %CD4+ | Inverse variance weighted | 32 | 0.028198 | 0.008508 | 0.000919 | 0.011522 | 0.044875 | 1.028599 | 1.011588 | 1.045897 | Promote |
| GCST90001585 | z79XSR | 30664745-GCST007090-EFO_0004616.h.tsv | CD14+ CD16+ monocyte %monocyte | Inverse variance weighted | 25 | 0.036169 | 0.016536 | 0.028721 | 0.003759 | 0.068579 | 1.036831 | 1.003766 | 1.070985 | Promote |
| GCST90001789 | z79XSR | 30664745-GCST007090-EFO_0004616.h.tsv | CD25 on IgD- CD38dim | Inverse variance weighted | 23 | 0.024514 | 0.011725 | 0.036562 | 0.001532 | 0.047496 | 1.024816 | 1.001533 | 1.048641 | Promote |
| GCST90001778 | z79XSR | 30664745-GCST007090-EFO_0004616.h.tsv | CD25 on IgD+ CD24+ | Inverse variance weighted | 27 | 0.016453 | 0.008191 | 0.044583 | 0.000398 | 0.032508 | 1.016589 | 1.000398 | 1.033043 | Promote |
| GCST90001796 | z79XSR | 30664745-GCST007090-EFO_0004616.h.tsv | CD27 on CD20- | Inverse variance weighted | 16 | 0.027045 | 0.013404 | 0.043617 | 0.000774 | 0.053316 | 1.027414 | 1.000774 | 1.054763 | Promote |
| GCST90001798 | z79XSR | 30664745-GCST007090-EFO_0004616.h.tsv | CD27 on CD24+ CD27+ | Inverse variance weighted | 31 | 0.018111 | 0.008851 | 0.040748 | 0.000762 | 0.03546 | 1.018276 | 1.000762 | 1.036096 | Promote |
| GCST90001528 | z79XSR | 30664745-GCST007090-EFO_0004616.h.tsv | CD33dim HLA DR+ CD11b- %CD33dim HLA DR+ | Inverse variance weighted | 25 | 0.019908 | 0.009133 | 0.029273 | 0.002007 | 0.037809 | 1.020108 | 1.002009 | 1.038533 | Promote |
| GCST90002053 | z79XSR | 30664745-GCST007090-EFO_0004616.h.tsv | CD45 on CD33br HLA DR+ | Inverse variance weighted | 16 | 0.018946 | 0.008064 | 0.018797 | 0.003141 | 0.034751 | 1.019127 | 1.003146 | 1.035362 | Promote |
| GCST90001915 | z79XSR | 30664745-GCST007090-EFO_0004616.h.tsv | CD45 on T cell | Inverse variance weighted | 15 | 0.02495 | 0.010066 | 0.013189 | 0.005221 | 0.04468 | 1.025264 | 1.005234 | 1.045693 | Promote |
| GCST90001904 | z79XSR | 30664745-GCST007090-EFO_0004616.h.tsv | CD86 on CD62L+ myeloid DC | Inverse variance weighted | 23 | 0.026113 | 0.012583 | 0.037966 | 0.00145 | 0.050776 | 1.026457 | 1.001451 | 1.052087 | Promote |
| GCST90001424 | z79XSR | 30664745-GCST007090-EFO_0004616.h.tsv | IgD+ %Lymphocyte | Inverse variance weighted | 19 | 0.025768 | 0.010386 | 0.013103 | 0.005411 | 0.046124 | 1.026102 | 1.005425 | 1.047205 | Promote |
| GCST90001515 | z79XSR | 30664745-GCST007090-EFO_0004616.h.tsv | Im MDSC AC | Inverse variance weighted | 23 | 0.022731 | 0.009766 | 0.019928 | 0.003591 | 0.041872 | 1.022992 | 1.003597 | 1.042761 | Promote |
| GCST90001434 | z79XSR | 30664745-GCST007090-EFO_0004616.h.tsv | Sw mem %lymphocyte | Inverse variance weighted | 18 | 0.0368 | 0.016631 | 0.026918 | 0.004203 | 0.069397 | 1.037485 | 1.004212 | 1.071861 | Promote |
| GCST90001617 | z79XSR | 30664745-GCST007090-EFO_0004616.h.tsv | TCRgd %lymphocyte | Inverse variance weighted | 21 | 0.01856 | 0.009412 | 0.048618 | 0.000112 | 0.037008 | 1.018733 | 1.000112 | 1.037701 | Promote |
| GCST90001615 | z79XSR | 30664745-GCST007090-EFO_0004616.h.tsv | TCRgd AC | Inverse variance weighted | 21 | 0.028216 | 0.014384 | 0.0498 | 2.41E-05 | 0.056408 | 1.028618 | 1.000024 | 1.05803 | Promote |

**Supplementary Table 5.** Mendelian randomization analysis results between metabolites and osteoarthritis.

| **id** | **nsnp** | **method** | **b** | **se** | **or** | **or_lci95** | **or_uci95** | **pvalue** |
| --- | --- | --- | --- | --- | --- | --- | --- | --- |
| GCST90199836 | 22 | MR Egger | 0.003152 | 0.064957 | 1.003157 | 0.883234 | 1.139361 | 0.961784 |
| GCST90199836 | 22 | Weighted median | 0.055327 | 0.039397 | 1.056887 | 0.978346 | 1.141732 | 0.160217 |
| GCST90199836 | 22 | Inverse variance weighted | 0.066296 | 0.029235 | 1.068543 | 1.009036 | 1.13156 | 0.023347 |
| GCST90199836 | 22 | Simple mode | 0.086639 | 0.076483 | 1.090503 | 0.938693 | 1.266865 | 0.270068 |
| GCST90199836 | 22 | Weighted mode | 0.070277 | 0.076678 | 1.072805 | 0.923105 | 1.246783 | 0.369801 |
| GCST90199839 | 42 | MR Egger | -0.03428 | 0.03094 | 0.966296 | 0.90944 | 1.026707 | 0.27443 |
| GCST90199839 | 42 | Weighted median | -0.04137 | 0.024599 | 0.959479 | 0.914316 | 1.006873 | 0.092653 |
| GCST90199839 | 42 | Inverse variance weighted | -0.05184 | 0.016324 | 0.949478 | 0.919581 | 0.980347 | 0.001494 |
| GCST90199839 | 42 | Simple mode | -0.04478 | 0.04113 | 0.956206 | 0.882148 | 1.036482 | 0.282606 |
| GCST90199839 | 42 | Weighted mode | -0.04122 | 0.0267 | 0.95962 | 0.910692 | 1.011177 | 0.13034 |
| GCST90200116 | 30 | MR Egger | 0.031209 | 0.062306 | 1.031701 | 0.913099 | 1.165707 | 0.62036 |
| GCST90200116 | 30 | Weighted median | 0.05565 | 0.033886 | 1.057228 | 0.989292 | 1.129829 | 0.100529 |
| GCST90200116 | 30 | Inverse variance weighted | 0.051973 | 0.026051 | 1.053347 | 1.000915 | 1.108527 | 0.046033 |
| GCST90200116 | 30 | Simple mode | 0.075135 | 0.060367 | 1.07803 | 0.957734 | 1.213436 | 0.223231 |
| GCST90200116 | 30 | Weighted mode | 0.06276 | 0.046849 | 1.064771 | 0.971355 | 1.167171 | 0.190768 |
| GCST90200145 | 33 | MR Egger | 0.076051 | 0.050805 | 1.079018 | 0.976747 | 1.191996 | 0.144529 |
| GCST90200145 | 33 | Weighted median | 0.043496 | 0.029827 | 1.044455 | 0.985147 | 1.107334 | 0.144762 |
| GCST90200145 | 33 | Inverse variance weighted | 0.044832 | 0.021484 | 1.045852 | 1.002726 | 1.090833 | 0.036913 |
| GCST90200145 | 33 | Simple mode | 0.095809 | 0.055071 | 1.100548 | 0.987942 | 1.22599 | 0.09152 |
| GCST90200145 | 33 | Weighted mode | 0.06185 | 0.034077 | 1.063803 | 0.995071 | 1.137283 | 0.078907 |
| GCST90200153 | 36 | MR Egger | 0.041618 | 0.033736 | 1.042496 | 0.975793 | 1.113758 | 0.225804 |
| GCST90200153 | 36 | Weighted median | 0.017773 | 0.02837 | 1.017932 | 0.962874 | 1.076138 | 0.531002 |
| GCST90200153 | 36 | Inverse variance weighted | 0.048338 | 0.01792 | 1.049525 | 1.013302 | 1.087043 | 0.006988 |
| GCST90200153 | 36 | Simple mode | -0.00169 | 0.050556 | 0.99831 | 0.90413 | 1.1023 | 0.973497 |
| GCST90200153 | 36 | Weighted mode | 0.001616 | 0.045137 | 1.001617 | 0.916813 | 1.094266 | 0.97165 |
| GCST90200172 | 26 | MR Egger | 0.064897 | 0.047407 | 1.067049 | 0.972368 | 1.17095 | 0.183699 |
| GCST90200172 | 26 | Weighted median | 0.056358 | 0.032548 | 1.057976 | 0.992592 | 1.127667 | 0.083356 |
| GCST90200172 | 26 | Inverse variance weighted | 0.077191 | 0.023683 | 1.080249 | 1.031252 | 1.131574 | 0.001116 |
| GCST90200172 | 26 | Simple mode | 0.070198 | 0.056687 | 1.07272 | 0.959917 | 1.19878 | 0.227092 |
| GCST90200172 | 26 | Weighted mode | 0.052449 | 0.040783 | 1.053849 | 0.97289 | 1.141546 | 0.210203 |
| GCST90200341 | 23 | MR Egger | 0.052554 | 0.081924 | 1.053959 | 0.897613 | 1.237538 | 0.528138 |
| GCST90200341 | 23 | Weighted median | 0.04945 | 0.035327 | 1.050693 | 0.980404 | 1.126022 | 0.161579 |
| GCST90200341 | 23 | Inverse variance weighted | 0.06727 | 0.029134 | 1.069584 | 1.01022 | 1.132437 | 0.020943 |
| GCST90200341 | 23 | Simple mode | 0.106528 | 0.066496 | 1.11241 | 0.976478 | 1.267263 | 0.123409 |
| GCST90200341 | 23 | Weighted mode | 0.033304 | 0.04946 | 1.033865 | 0.938345 | 1.139108 | 0.507735 |
| GCST90200347 | 26 | MR Egger | -0.12571 | 0.058143 | 0.881875 | 0.786891 | 0.988324 | 0.040808 |
| GCST90200347 | 26 | Weighted median | -0.03511 | 0.03602 | 0.965498 | 0.899684 | 1.036126 | 0.329676 |
| GCST90200347 | 26 | Inverse variance weighted | -0.05411 | 0.027485 | 0.947325 | 0.897643 | 0.999757 | 0.048971 |
| GCST90200347 | 26 | Simple mode | -0.16791 | 0.084061 | 0.845433 | 0.71701 | 0.996859 | 0.056772 |
| GCST90200347 | 26 | Weighted mode | -0.16791 | 0.084598 | 0.845433 | 0.716256 | 0.997907 | 0.058248 |
| GCST90200608 | 27 | MR Egger | 0.02388 | 0.024999 | 1.024168 | 0.975195 | 1.0756 | 0.348601 |
| GCST90200608 | 27 | Weighted median | 0.032611 | 0.023367 | 1.033148 | 0.986897 | 1.081567 | 0.162843 |
| GCST90200608 | 27 | Inverse variance weighted | 0.036739 | 0.018217 | 1.037422 | 1.001034 | 1.075133 | 0.043722 |
| GCST90200608 | 27 | Simple mode | 0.023994 | 0.052136 | 1.024285 | 0.924787 | 1.134486 | 0.649178 |
| GCST90200608 | 27 | Weighted mode | 0.033259 | 0.024784 | 1.033819 | 0.984799 | 1.085278 | 0.191213 |
| GCST90200621 | 28 | MR Egger | 0.06484 | 0.037481 | 1.066988 | 0.991413 | 1.148323 | 0.095502 |
| GCST90200621 | 28 | Weighted median | 0.037966 | 0.031913 | 1.038695 | 0.975716 | 1.105741 | 0.234182 |
| GCST90200621 | 28 | Inverse variance weighted | 0.04861 | 0.020196 | 1.049811 | 1.009066 | 1.092201 | 0.016089 |
| GCST90200621 | 28 | Simple mode | 0.073952 | 0.051136 | 1.076755 | 0.974067 | 1.190268 | 0.159639 |
| GCST90200621 | 28 | Weighted mode | 0.041752 | 0.031934 | 1.042636 | 0.979378 | 1.10998 | 0.202077 |
| GCST90200639 | 22 | MR Egger | 0.091572 | 0.059926 | 1.095895 | 0.97445 | 1.232477 | 0.142152 |
| GCST90200639 | 22 | Weighted median | 0.061741 | 0.034353 | 1.063687 | 0.994425 | 1.137774 | 0.072295 |
| GCST90200639 | 22 | Inverse variance weighted | 0.066931 | 0.023645 | 1.069222 | 1.0208 | 1.119941 | 0.004645 |
| GCST90200639 | 22 | Simple mode | 0.062625 | 0.066859 | 1.064628 | 0.93387 | 1.213693 | 0.35957 |
| GCST90200639 | 22 | Weighted mode | 0.059773 | 0.064672 | 1.061596 | 0.93521 | 1.205062 | 0.365854 |
| GCST90200971 | 26 | MR Egger | 0.166039 | 0.069416 | 1.180619 | 1.030438 | 1.352689 | 0.024945 |
| GCST90200971 | 26 | Weighted median | 0.063055 | 0.040748 | 1.065085 | 0.983329 | 1.153639 | 0.12176 |
| GCST90200971 | 26 | Inverse variance weighted | 0.075905 | 0.029568 | 1.07886 | 1.018113 | 1.14323 | 0.010255 |
| GCST90200971 | 26 | Simple mode | 0.13295 | 0.089087 | 1.142192 | 0.959194 | 1.360104 | 0.148121 |
| GCST90200971 | 26 | Weighted mode | 0.126936 | 0.088412 | 1.135344 | 0.954707 | 1.35016 | 0.163472 |

**Supplementary Table 6.** Mendelian randomization analysis results between core genes and immune cells.

| **id** | **nsnp** | **method** | **b** | **se** | **or** | **or_lci95** | **or_uci95** | **pvalue** |
| --- | --- | --- | --- | --- | --- | --- | --- | --- |
| GCST90001424 | 26 | MR Egger | -0.01049 | 0.098881 | 0.989561 | 0.815217 | 1.201192 | 0.916367 |
| GCST90001424 | 26 | Weighted median | 0.037883 | 0.061561 | 1.03861 | 0.920557 | 1.171802 | 0.538307 |
| GCST90001424 | 26 | Inverse variance weighted | 0.040084 | 0.050923 | 1.040898 | 0.942024 | 1.15015 | 0.431196 |
| GCST90001424 | 26 | Simple mode | 0.054496 | 0.120003 | 1.056009 | 0.834678 | 1.33603 | 0.653656 |
| GCST90001424 | 26 | Weighted mode | 0.043869 | 0.055567 | 1.044846 | 0.937028 | 1.165069 | 0.437255 |
| GCST90001434 | 26 | MR Egger | 0.101245 | 0.093376 | 1.106547 | 0.921482 | 1.32878 | 0.289018 |
| GCST90001434 | 26 | Weighted median | 0.011608 | 0.066541 | 1.011675 | 0.887974 | 1.152609 | 0.861516 |
| GCST90001434 | 26 | Inverse variance weighted | -0.05172 | 0.048709 | 0.949598 | 0.863133 | 1.044725 | 0.288353 |
| GCST90001434 | 26 | Simple mode | 0.046486 | 0.118331 | 1.047583 | 0.830736 | 1.321034 | 0.697765 |
| GCST90001434 | 26 | Weighted mode | 0.026052 | 0.057993 | 1.026395 | 0.916115 | 1.149949 | 0.657131 |
| GCST90001500 | 26 | MR Egger | -0.12225 | 0.092448 | 0.884925 | 0.738266 | 1.060718 | 0.198503 |
| GCST90001500 | 26 | Weighted median | -0.02257 | 0.065746 | 0.977682 | 0.859475 | 1.112147 | 0.731376 |
| GCST90001500 | 26 | Inverse variance weighted | -0.05179 | 0.048303 | 0.949527 | 0.863756 | 1.043816 | 0.283624 |
| GCST90001500 | 26 | Simple mode | -0.02488 | 0.121395 | 0.975426 | 0.768885 | 1.23745 | 0.839265 |
| GCST90001500 | 26 | Weighted mode | -0.01303 | 0.061312 | 0.987058 | 0.875291 | 1.113095 | 0.833464 |
| GCST90001515 | 26 | MR Egger | -0.16322 | 0.125174 | 0.849408 | 0.664608 | 1.085593 | 0.204634 |
| GCST90001515 | 26 | Weighted median | -0.06776 | 0.094958 | 0.934486 | 0.775788 | 1.125648 | 0.475499 |
| GCST90001515 | 26 | Inverse variance weighted | -0.02314 | 0.065387 | 0.977128 | 0.859593 | 1.110735 | 0.723451 |
| GCST90001515 | 26 | Simple mode | 0.219505 | 0.166529 | 1.24546 | 0.898624 | 1.726164 | 0.199413 |
| GCST90001515 | 26 | Weighted mode | -0.06098 | 0.084244 | 0.94084 | 0.797638 | 1.109751 | 0.475859 |
| GCST90001528 | 26 | MR Egger | -0.08475 | 0.149348 | 0.918741 | 0.685592 | 1.231176 | 0.57566 |
| GCST90001528 | 26 | Weighted median | -0.00408 | 0.092587 | 0.995927 | 0.830646 | 1.194095 | 0.96484 |
| GCST90001528 | 26 | Inverse variance weighted | -0.00344 | 0.077075 | 0.996569 | 0.856841 | 1.159084 | 0.964436 |
| GCST90001528 | 26 | Simple mode | 0.040886 | 0.164216 | 1.041733 | 0.755046 | 1.437275 | 0.805414 |
| GCST90001528 | 26 | Weighted mode | 0.018117 | 0.09651 | 1.018282 | 0.842786 | 1.230322 | 0.852614 |
| GCST90001585 | 26 | MR Egger | 0.120973 | 0.092892 | 1.128594 | 0.940733 | 1.35397 | 0.205176 |
| GCST90001585 | 26 | Weighted median | 0.196745 | 0.068723 | 1.217433 | 1.064012 | 1.392976 | 0.004199 |
| GCST90001585 | 26 | Inverse variance weighted | 0.182108 | 0.048465 | 1.199744 | 1.091024 | 1.319299 | 0.000172 |
| GCST90001585 | 26 | Simple mode | 0.103758 | 0.116619 | 1.109332 | 0.88266 | 1.394216 | 0.3821 |
| GCST90001585 | 26 | Weighted mode | 0.112816 | 0.069751 | 1.119426 | 0.976387 | 1.283419 | 0.118341 |
| GCST90001615 | 26 | MR Egger | 0.000494 | 0.091341 | 1.000494 | 0.836496 | 1.196645 | 0.995727 |
| GCST90001615 | 26 | Weighted median | -0.00456 | 0.065088 | 0.995451 | 0.876226 | 1.130899 | 0.944158 |
| GCST90001615 | 26 | Inverse variance weighted | -0.00667 | 0.047709 | 0.993349 | 0.904672 | 1.090719 | 0.888766 |
| GCST90001615 | 26 | Simple mode | 0.136868 | 0.117801 | 1.146677 | 0.910263 | 1.444494 | 0.256266 |
| GCST90001615 | 26 | Weighted mode | -0.00375 | 0.062141 | 0.996254 | 0.882012 | 1.125292 | 0.952317 |
| GCST90001617 | 26 | MR Egger | 0.026279 | 0.093113 | 1.026627 | 0.855369 | 1.232173 | 0.780192 |
| GCST90001617 | 26 | Weighted median | -0.00142 | 0.068788 | 0.998582 | 0.872631 | 1.142713 | 0.983541 |
| GCST90001617 | 26 | Inverse variance weighted | -0.02473 | 0.04862 | 0.975577 | 0.886902 | 1.073118 | 0.611057 |
| GCST90001617 | 26 | Simple mode | 0.120454 | 0.114978 | 1.128009 | 0.900413 | 1.413134 | 0.304833 |
| GCST90001617 | 26 | Weighted mode | 0.013474 | 0.064414 | 1.013565 | 0.89335 | 1.149958 | 0.836005 |
| GCST90001778 | 26 | MR Egger | 0.090386 | 0.091731 | 1.094597 | 0.914473 | 1.3102 | 0.334289 |
| GCST90001778 | 26 | Weighted median | 0.045669 | 0.067283 | 1.046728 | 0.917405 | 1.194281 | 0.497293 |
| GCST90001778 | 26 | Inverse variance weighted | 0.02202 | 0.047862 | 1.022264 | 0.930727 | 1.122805 | 0.645462 |
| GCST90001778 | 26 | Simple mode | -0.06661 | 0.108292 | 0.935556 | 0.75664 | 1.156778 | 0.544026 |
| GCST90001778 | 26 | Weighted mode | 0.045686 | 0.061335 | 1.046746 | 0.928179 | 1.180458 | 0.463304 |
| GCST90001789 | 26 | MR Egger | 0.091679 | 0.106171 | 1.096012 | 0.890105 | 1.349553 | 0.396409 |
| GCST90001789 | 26 | Weighted median | 0.13812 | 0.066894 | 1.148114 | 1.007033 | 1.308959 | 0.038945 |
| GCST90001789 | 26 | Inverse variance weighted | 0.115457 | 0.054357 | 1.122386 | 1.008958 | 1.248567 | 0.033664 |
| GCST90001789 | 26 | Simple mode | 0.117655 | 0.103092 | 1.124855 | 0.919059 | 1.376735 | 0.264576 |
| GCST90001789 | 26 | Weighted mode | 0.127706 | 0.055825 | 1.136218 | 1.018457 | 1.267596 | 0.03089 |
| GCST90001796 | 26 | MR Egger | -0.05996 | 0.103483 | 0.941803 | 0.768906 | 1.153577 | 0.56771 |
| GCST90001796 | 26 | Weighted median | -0.06148 | 0.060644 | 0.940374 | 0.834987 | 1.059062 | 0.310703 |
| GCST90001796 | 26 | Inverse variance weighted | -0.08939 | 0.053052 | 0.914485 | 0.824172 | 1.014695 | 0.091983 |
| GCST90001796 | 26 | Simple mode | -0.18983 | 0.110832 | 0.827101 | 0.665605 | 1.027782 | 0.099136 |
| GCST90001796 | 26 | Weighted mode | -0.05647 | 0.061382 | 0.945097 | 0.837966 | 1.065924 | 0.366399 |
| GCST90001798 | 26 | MR Egger | 0.063295 | 0.092115 | 1.065341 | 0.889362 | 1.27614 | 0.498589 |
| GCST90001798 | 26 | Weighted median | 0.084854 | 0.063872 | 1.088558 | 0.960467 | 1.233731 | 0.184015 |
| GCST90001798 | 26 | Inverse variance weighted | 0.097586 | 0.04806 | 1.102506 | 1.003395 | 1.211408 | 0.042304 |
| GCST90001798 | 26 | Simple mode | 0.074952 | 0.095041 | 1.077833 | 0.894645 | 1.29853 | 0.437744 |
| GCST90001798 | 26 | Weighted mode | 0.052885 | 0.05935 | 1.054308 | 0.93853 | 1.184369 | 0.381389 |
| GCST90001904 | 26 | MR Egger | 0.066268 | 0.103786 | 1.068513 | 0.871836 | 1.309557 | 0.529194 |
| GCST90001904 | 26 | Weighted median | 0.181747 | 0.068445 | 1.199311 | 1.048746 | 1.371491 | 0.007922 |
| GCST90001904 | 26 | Inverse variance weighted | 0.156149 | 0.05429 | 1.169 | 1.050998 | 1.300252 | 0.004025 |
| GCST90001904 | 26 | Simple mode | 0.191575 | 0.108574 | 1.211156 | 0.978994 | 1.498374 | 0.08987 |
| GCST90001904 | 26 | Weighted mode | 0.18292 | 0.063386 | 1.200719 | 1.060439 | 1.359555 | 0.007931 |
| GCST90001915 | 26 | MR Egger | 0.14513 | 0.098907 | 1.15619 | 0.95244 | 1.403528 | 0.155268 |
| GCST90001915 | 26 | Weighted median | 0.095115 | 0.075536 | 1.099785 | 0.948441 | 1.27528 | 0.207959 |
| GCST90001915 | 26 | Inverse variance weighted | 0.020634 | 0.052869 | 1.020848 | 0.92036 | 1.132307 | 0.696333 |
| GCST90001915 | 26 | Simple mode | 0.067274 | 0.121455 | 1.069589 | 0.843008 | 1.357068 | 0.584568 |
| GCST90001915 | 26 | Weighted mode | 0.082289 | 0.071508 | 1.08577 | 0.943777 | 1.249126 | 0.260712 |
| GCST90002053 | 26 | MR Egger | 0.18212 | 0.137956 | 1.199758 | 0.915511 | 1.572257 | 0.199244 |
| GCST90002053 | 26 | Weighted median | 0.184473 | 0.097241 | 1.202584 | 0.993898 | 1.455087 | 0.05782 |
| GCST90002053 | 26 | Inverse variance weighted | 0.164109 | 0.072016 | 1.178343 | 1.023224 | 1.356977 | 0.022679 |
| GCST90002053 | 26 | Simple mode | 0.125019 | 0.154027 | 1.133171 | 0.837886 | 1.532518 | 0.42464 |
| GCST90002053 | 26 | Weighted mode | 0.158992 | 0.097812 | 1.172329 | 0.96781 | 1.420068 | 0.116602 |

**Supplementary Table 7.** Mendelian randomization analysis results between core genes and metabolites.

| **id** | **nsnp** | **method** | **b** | **se** | **or** | **or_lci95** | **or_uci95** | **pvalue** |
| --- | --- | --- | --- | --- | --- | --- | --- | --- |
| GCST90199836 | 25 | MR Egger | 0.041074 | 0.064055 | 1.041929 | 0.918996 | 1.181307 | 0.527714 |
| GCST90199836 | 25 | Weighted median | -0.05237 | 0.048281 | 0.948981 | 0.863297 | 1.04317 | 0.27809 |
| GCST90199836 | 25 | Inverse variance weighted | -0.0246 | 0.033308 | 0.975699 | 0.914037 | 1.041522 | 0.460158 |
| GCST90199836 | 25 | Simple mode | -0.07806 | 0.085172 | 0.924911 | 0.782709 | 1.092949 | 0.368538 |
| GCST90199836 | 25 | Weighted mode | -0.03288 | 0.050638 | 0.967658 | 0.87623 | 1.068625 | 0.522341 |
| GCST90200116 | 25 | MR Egger | -0.03071 | 0.067071 | 0.969752 | 0.850293 | 1.105995 | 0.651294 |
| GCST90200116 | 25 | Weighted median | -0.10829 | 0.046 | 0.897368 | 0.820002 | 0.982034 | 0.018566 |
| GCST90200116 | 25 | Inverse variance weighted | -0.12179 | 0.034563 | 0.885337 | 0.827348 | 0.947391 | 0.000426 |
| GCST90200116 | 25 | Simple mode | -0.13992 | 0.07105 | 0.869431 | 0.756408 | 0.999342 | 0.060572 |
| GCST90200116 | 25 | Weighted mode | -0.11845 | 0.046761 | 0.888294 | 0.8105 | 0.973555 | 0.018253 |
| GCST90200145 | 25 | MR Egger | -0.04814 | 0.062176 | 0.953004 | 0.843663 | 1.076515 | 0.446702 |
| GCST90200145 | 25 | Weighted median | -0.04101 | 0.047436 | 0.959817 | 0.874601 | 1.053337 | 0.38727 |
| GCST90200145 | 25 | Inverse variance weighted | -0.03904 | 0.032049 | 0.961717 | 0.903163 | 1.024066 | 0.223229 |
| GCST90200145 | 25 | Simple mode | -0.01266 | 0.074769 | 0.987416 | 0.852815 | 1.14326 | 0.866918 |
| GCST90200145 | 25 | Weighted mode | -0.01916 | 0.045345 | 0.981025 | 0.897598 | 1.072208 | 0.676445 |
| GCST90200153 | 25 | MR Egger | 0.039266 | 0.070529 | 1.040047 | 0.905769 | 1.194232 | 0.583084 |
| GCST90200153 | 25 | Weighted median | 0.056167 | 0.048636 | 1.057775 | 0.961598 | 1.163571 | 0.24815 |
| GCST90200153 | 25 | Inverse variance weighted | 0.029057 | 0.035613 | 1.029484 | 0.960074 | 1.103911 | 0.414549 |
| GCST90200153 | 25 | Simple mode | 0.100786 | 0.092921 | 1.10604 | 0.921882 | 1.326986 | 0.288855 |
| GCST90200153 | 25 | Weighted mode | 0.061786 | 0.055793 | 1.063734 | 0.953545 | 1.186657 | 0.279094 |
| GCST90200172 | 25 | MR Egger | 0.022228 | 0.062387 | 1.022476 | 0.904791 | 1.15547 | 0.724876 |
| GCST90200172 | 25 | Weighted median | 0.034725 | 0.044077 | 1.035335 | 0.949645 | 1.128756 | 0.430807 |
| GCST90200172 | 25 | Inverse variance weighted | 0.014054 | 0.032156 | 1.014153 | 0.952208 | 1.080128 | 0.662077 |
| GCST90200172 | 25 | Simple mode | 0.027357 | 0.0732 | 1.027735 | 0.890374 | 1.186288 | 0.711881 |
| GCST90200172 | 25 | Weighted mode | 0.034851 | 0.041811 | 1.035465 | 0.953993 | 1.123896 | 0.412767 |
| GCST90200341 | 25 | MR Egger | -0.05295 | 0.064985 | 0.948423 | 0.834999 | 1.077254 | 0.4235 |
| GCST90200341 | 25 | Weighted median | -0.02808 | 0.04574 | 0.97231 | 0.888935 | 1.063504 | 0.539263 |
| GCST90200341 | 25 | Inverse variance weighted | -0.05246 | 0.032789 | 0.948895 | 0.889831 | 1.01188 | 0.109635 |
| GCST90200341 | 25 | Simple mode | -0.02239 | 0.06858 | 0.977863 | 0.854872 | 1.118548 | 0.746936 |
| GCST90200341 | 25 | Weighted mode | -0.03374 | 0.04137 | 0.966821 | 0.89152 | 1.048483 | 0.422751 |
| GCST90200608 | 25 | MR Egger | -0.00745 | 0.059418 | 0.992582 | 0.883464 | 1.115176 | 0.901364 |
| GCST90200608 | 25 | Weighted median | -0.03199 | 0.042898 | 0.968514 | 0.89041 | 1.053468 | 0.455799 |
| GCST90200608 | 25 | Inverse variance weighted | -0.00032 | 0.030626 | 0.999677 | 0.941436 | 1.061521 | 0.99158 |
| GCST90200608 | 25 | Simple mode | -0.02508 | 0.077698 | 0.975233 | 0.837472 | 1.135655 | 0.749663 |
| GCST90200608 | 25 | Weighted mode | -0.02791 | 0.045775 | 0.972476 | 0.889027 | 1.063759 | 0.547782 |
| GCST90200621 | 25 | MR Egger | -0.07032 | 0.065924 | 0.932092 | 0.819111 | 1.060656 | 0.297157 |
| GCST90200621 | 25 | Weighted median | 0.000776 | 0.047589 | 1.000777 | 0.911651 | 1.098615 | 0.986982 |
| GCST90200621 | 25 | Inverse variance weighted | 0.014955 | 0.034872 | 1.015067 | 0.948005 | 1.086873 | 0.668039 |
| GCST90200621 | 25 | Simple mode | -0.04953 | 0.087769 | 0.951681 | 0.801273 | 1.130322 | 0.577808 |
| GCST90200621 | 25 | Weighted mode | -0.02217 | 0.044663 | 0.978071 | 0.896091 | 1.067551 | 0.624101 |
| GCST90200639 | 25 | MR Egger | -0.14257 | 0.072646 | 0.867128 | 0.752048 | 0.999818 | 0.061912 |
| GCST90200639 | 25 | Weighted median | -0.03524 | 0.053212 | 0.96537 | 0.869759 | 1.071492 | 0.507764 |
| GCST90200639 | 25 | Inverse variance weighted | -0.04192 | 0.037437 | 0.958947 | 0.891102 | 1.031958 | 0.262837 |
| GCST90200639 | 25 | Simple mode | 0.013753 | 0.086337 | 1.013848 | 0.856015 | 1.200782 | 0.874772 |
| GCST90200639 | 25 | Weighted mode | -0.04211 | 0.050343 | 0.958761 | 0.868675 | 1.058189 | 0.411107 |
| GCST90200971 | 25 | MR Egger | -0.04689 | 0.058145 | 0.954196 | 0.851419 | 1.06938 | 0.428297 |
| GCST90200971 | 25 | Weighted median | -0.0501 | 0.04344 | 0.951136 | 0.873506 | 1.035665 | 0.248794 |
| GCST90200971 | 25 | Inverse variance weighted | -0.05398 | 0.029966 | 0.947455 | 0.89341 | 1.004769 | 0.071665 |
| GCST90200971 | 25 | Simple mode | -0.02467 | 0.077764 | 0.975627 | 0.837703 | 1.136261 | 0.75376 |
| GCST90200971 | 25 | Weighted mode | -0.05406 | 0.042431 | 0.947375 | 0.871775 | 1.029531 | 0.214832 |

**Supplementary Table 8:** Binding energies of 13 candidate drugs.

| GW589933X | 4-[[(Z)-(7-oxo-6H-pyrrolo[2,3-g][1,3]benzothiazol-8-ylidene)methyl]amino]benzenesulfonamide | -8.3 kcal/mol |
| --- | --- | --- |
| GW612286X | N~4~-(3-methyl-1H-indazol-6-yl)-N~2~-(3,4,5-trimethoxyphenyl)pyrimidine-2,4-diamine | -8.2 kcal/mol |
| GW770249A | 1-[4-(4-Aminofuro[2,3-d]pyrimidin-5-yl)phenyl]-3-[2-fluoro-5-(trifluoromethyl)phenyl]urea;hydrochloride | -1.0 kcal/mol |
| GW795493X | 3-(4-{4-Aminofuro[2,3-d]pyrimidin-5-yl}phenyl)-1-[3-(trifluoromethyl)phenyl]urea | -9.7 kcal/mol |
| GW810576X | Pyrazolopyridazine 1 | -7.8 kcal/mol |
| Kinome_439 | Urea, N-[4-[7-amino-3-(1-methyl-1H-pyrazol-4-yl)pyrazolo[1,5-a]pyrimidin-6-yl]phenyl]-N'-[3-(trifluoromethyl)phenyl]- | -10.1 kcal/mol |
| Kinome_450 | 1-[4-[4-Amino-7-[1-(2-hydroxy-2-methylpropyl)pyrazol-4-yl]thieno[3,2-c]pyridin-3-yl]phenyl]-3-(4-methoxyphenyl)urea | -8.7 kcal/mol |
| Kinome_474 | 4-amino-3-[4-[(2-fluoro-5-methylphenyl)carbamoylamino]phenyl]-N-methylthieno[3,2-c]pyridine-7-carboxamide | -9.1 kcal/mol |
| Kinome_490 | 1-[4-[4-Amino-7-(1-methylpyrazol-4-yl)thieno[3,2-c]pyridin-3-yl]phenyl]-3-(4-ethoxyphenyl)urea | -9.1 kcal/mol |
| Kinome_504 | 1-[4-[4-Amino-7-[1-(2-hydroxypropyl)pyrazol-4-yl]thieno[3,2-c]pyridin-3-yl]phenyl]-3-(3-fluorophenyl)urea | -8.8 kcal/mol |
| Kinome_506 | 1-[4-[4-Amino-7-[1-(2-hydroxyethyl)pyrazol-4-yl]thieno[3,2-c]pyridin-3-yl]phenyl]-3-[4-(difluoromethoxy)phenyl]urea | -8.3 kcal/mol |
| Kinome_2812 | 3-[4-amino-3-(7-fluoro-2-methyl-1H-indol-5-yl)thieno[3,2-c]pyridin-7-yl]-N-methylprop-2-enamide | -8.8 kcal/mol |
| PDK1/Akt/Flt Dual Pathway Inhibitor | PDK1/Akt/Flt Dual Pathway Inhibitor | -8.3 kcal/mol |
