# Supplementary material for: Virtual single‐cell perturbation and genetic causal inference reveal CSF1R‐dependent immunometabolic communication in iron metabolism‐associated osteoarthritis
Source: J Cell Commun Signal. 2026 Aug 27;20(3):e70107. doi: 10.1002/ccs3.70107 (PMC13519958; doi:10.1002/ccs3.70107)
Supplement: Supplementary file 2 [file CCS3-20-e70107-s006.docx]

**Supplementary Figure 1.** Mendelian randomization analysis of iron metabolism disorder and osteoarthritis. (a) Forest plot of the MR analysis. (b) Scatter plot of the MR analysis. (c) Funnel plot of the MR analysis.

**Supplementary Figure 2.** (a) Principal component analysis of the osteoarthritis dataset before batch effect correction; (b) PCA of the osteoarthritis dataset after batch effect correction; (c) Heatmap of differentially expressed genes in osteoarthritis; (d–f) ROC curves for the test cohort; (g–i) Confusion matrices of the test cohort.

**Supplementary Figure 3.** (a) Cumulative distribution function curves for consensus clustering with k ranging from 2 to 9; (b) Relative change in the area under the CDF curve for k = 2 to 9; (c) Comparison of expression levels of 11 key genes between the two subgroups; (d) Boxplot analysis of the relative abundance of immune cells between the two clusters; (e–t) Correlation analysis between the core gene CSF1R and various immune cells, all showing statistical significance.

**Supplementary Figure 4.** Upregulation of CSF1R protein expression in the disease group. (a) Representative Western blot bands of CSF1R protein expression in the control and disease groups. (b) Quantitative analysis of the relative protein expression level of CSF1R.
